# A bacterial endosymbiont of the fungus *Rhizopus microsporus* drives phagocyte evasion and opportunistic virulence

**DOI:** 10.1016/j.cub.2022.01.028

**Published:** 2022-03-14

**Authors:** Herbert Itabangi, Poppy C.S. Sephton-Clark, Diana P. Tamayo, Xin Zhou, Georgina P. Starling, Zamzam Mahamoud, Ignacio Insua, Mark Probert, Joao Correia, Patrick J. Moynihan, Teclegiorgis Gebremariam, Yiyou Gu, Ashraf S. Ibrahim, Gordon D. Brown, Jason S. King, Elizabeth R. Ballou, Kerstin Voelz

**Affiliations:** 1 Institute of Microbiology and Infection, School of Biosciences, University of Birmingham, Edgbaston, Birmingham B15 2TT, UK; 2 MRC Centre for Medical Mycology, University of Exeter, Geoffrey Pope Building, Stocker Road, Exeter, EX4 4QD, UK; 3 School of Biosciences, University of Sheffield, Western Bank, Sheffield, S10 2TN, UK; 4 School of Chemistry, University of Birmingham, Edgbaston, Birmingham, B15 2TT, UK; 5 The Lundquist Institute for Biomedical Innovation at Harbor-UCLA Medical Center, Torrance, CA, USA; 6 David Geffen School of Medicine, UCLA, Los Angeles, CA, USA

**Keywords:** *Murcomycete*, *Rhizopus*, *Ralstonia*, *Dictyostelium*, endosymbiosis, evolution, soil microbiology, fungal pathogenesis

## Abstract

Opportunistic infections by environmental fungi are a growing clinical problem, driven by an increasing population of people with immunocompromising conditions. Spores of the Mucorales order are ubiquitous in the environment but can also cause acute invasive infections in humans through germination and evasion of the mammalian host immune system. How they achieve this and the evolutionary drivers underlying the acquisition of virulence mechanisms are poorly understood. Here, we show that a clinical isolate of *Rhizopus microsporus* contains a *Ralstonia pickettii* bacterial endosymbiont required for virulence in both zebrafish and mice and that this endosymbiosis enables the secretion of factors that potently suppress growth of the soil amoeba *Dictyostelium discoideum*, as well as their ability to engulf and kill other microbes. As amoebas are natural environmental predators of both bacteria and fungi, we propose that this tri-kingdom interaction contributes to establishing endosymbiosis and the acquisition of anti-phagocyte activity. Importantly, we show that this activity also protects fungal spores from phagocytosis and clearance by human macrophages, and endosymbiont removal renders the fungal spores avirulent *in vivo*. Together, these findings describe a new role for a bacterial endosymbiont in *Rhizopus microsporus* pathogenesis in animals and suggest a mechanism of virulence acquisition through environmental interactions with amoebas.

## Highlights

• Bacterial endosymbionts protect fungal spores from phagocytes
• A secreted factor blocks growth and killing by environmental amoebas
• Endosymbionts improve fungal stress resistance
• Endosymbiosis also allows the evasion of vertebrate immune cells and virulence *in vivo*

## Teaser

How environmental fungi evolved virulence mechanisms to opportunistically infect humans is unclear. Itabangi et al. identify a tri-kingdom interaction, whereby a bacterial endosymbiont living within a fungus causes the generation of a secreted factor that blocks the predatory activity of amoebas and drives virulence in animal models.

## Introduction

Soil-dwelling fungi must evade predation by phagocytic amoebas and, similarly, pathogenic fungi evade host phagocytic cells that defend against infection.^1^^,^^2^ Interaction with amoebae has been proposed as an evolutionary training ground for pathogenic fungi, enabling them to resist the multifactorial stresses of the mammalian host.^3^ Mucoralean fungi such as soil-associated *Rhizopus microsporus*, which causes invasive mucormycosis, successfully establish in complex polymicrobial environments that include predatory amoebas. Yet, they lack common strategies fungi employ to evade predation and phagocytosis: *R. microsporus* fails to mask cell wall ligands in swollen spores, has a slow germination rate, and produces relatively low biomass.^4^^,^^5^ Mucorales resting spores also fail to elicit pro-inflammatory cytokine responses and do not induce strong phagocyte chemotaxis. Here, we investigate an as-yet unexplored aspect of *R. microsporus* pathogen biology: the impact of a bacterial endosymbiont on interaction with amoebas and host phagocytes.

Pathogenic Mucorales span multiple genera, with the *Rhizopus* genus causing almost half of all documented cases and high mortality in susceptible patient populations.^6^, ^7^, ^8^, ^9^, ^10^, ^11^, ^12^, ^13^ Infection occurs through inoculation with dormant, immunologically inert spores. Upon germination, these spores become metabolically active and begin to swell.^14^^,^^15^ Based on analogy to *Aspergillus* spores, frequently used as a model for Mucorales infectious processes, swelling is expected to reveal microbe associated molecular patterns (MAMPs) and induce increasing rates of phagocytosis which must be overcome by rapid hyphal extension.^16^^,^^17^ However, swollen spores in the *Rhizopus* genus are often no more readily phagocytosed than resting spores, and *R. microsporus* spores are phagocytosed at lower rates than other well studied fungal spores.^18^, ^19^, ^20^, ^21^ While in some species, larger spore size (12.3 μm) reduces phagocytosis, the small spore size of *R. microsporus* (5 μm, comparable with other well-phagocytosed particles) makes this unlikely to explain the observed reduced uptake.^22^^,^^23^

A common strategy fungi employ to evade phagocytosis is through masking cell wall ligands.^17^^,^^24^, ^25^, ^26^, ^27^, ^28^, ^29^ Two classic examples of this are the *Cryptococcus neoformans* capsule and the *Aspergillus* spore hydrophobin layer, which effectively block phagocytosis by preventing detection of MAMPs.^16^^,^^17^^,^^28^, ^29^, ^30^, ^31^ However, there is no evidence of hydrophobins in the *R. microsporus* genome, and evidence of cell wall remodeling that masks MAMPs is limited to the hyphal phase.^32^ Moreover, the slow germination rate and low biomass of *R. microsporus* are also predicted to reduce fungal virulence.^4^^,^^5^^,^^22^^,^^33^, ^34^, ^35^ Together, these differences suggest an alternate strategy available to *Rhizopus* for the evasion of phagocytosis during the early stages of germination.

Members of the Mucormycota, which includes the order Mucorales, are sometimes colonized or influenced by endosymbiotic bacteria.^36^^,^^37^ The soil-dwelling plant bacterial pathogen *Ralstonia solanacearum* produces a lipopeptide, ralsolamycin, that aids invasion of fungal hypha and can influence chlamydospore development across a wide range of fungi, including members of the Mucoromycota, as well as Ascomycota and Basidiomycota.^38^^,^^39^ Bacteria of the genera *Mycetohabitans* (formerly *Burkholderia rhizoxinica* and *B. endofungorum*) are well-established endosymbionts of *R. microsporus* and have been identified in patient isolates.^40^, ^41^, ^42^ During endosymbiosis, *Mycetohabitans* spp. influence fungal behavior such as sporulation and mating.^43^, ^44^, ^45^ Endosymbiosis is also implicated in plant pathogenesis: the endotoxin rhizoxin is secreted by *Mycetohabitans rhizoxina* during mutualism with *R. microsporus* and inhibits plant defenses.^42^^,^^46^^,^^47^ While endosymbionts are widespread in patient fungal samples, a role for endosymbionts in preventing fungal phagocytosis has not been established.^40^^,^^48^ Rather, work examining this interaction in the context of phagocyte-deficient mouse models found no correlation between endosymbiont presence and fungal virulence.^40^ We recently showed that phagocyte activity in the early stages of infection control is critical for predicting Mucorales disease outcome, suggesting that phagocyte deficiency primes the host for infection.^49^ Together, this raises the hypothesis: do endosymbionts specifically impact fungal interaction with phagocytic cells, including both environmental amoebae and mammalian macrophages and neutrophils, leading to enhanced fungal virulence?

Here, we investigate the interaction of environmental and host phagocytic cells with *R. microsporus*. We report, for the first time, a role for a bacterial endosymbiont of *R. microsporus* in modulating the interaction with these phagocytic cells and further expand this to demonstrate a role in the early stages of disease in both zebrafish and murine models of infection. Specifically, we observed a significant reduction in phagocytosis of metabolically activated spores compared with resting spores. We investigate the consequences of endosymbiont status on infection outcome and demonstrate that this bacterial endosymbiont contributes to both fungal stress resistance and immune evasion during the earliest stages of infection, enabling fungal pathogenesis.

## Results

### A clinical isolate of *R. microsporus* suppresses phagocytosis by macrophages

We previously showed that the early stages of host-fungus interaction determine disease outcome in the zebrafish model of mucormycosis.^49^ Successful control of infection therefore requires both the presence of phagocytes at the site of infection within the first 24 h and their subsequent ability to kill spores. We hypothesized that, in instances where infection control fails, spores might evade phagocytosis. We therefore examined in detail the interactions between sporangiospores from *R. microsporus* strain FP469-12, a clinical isolate from a patient at the Queen Elizabeth Hospital, Birmingham, and J774A.1 macrophage-like cells.

**Figure 1:**
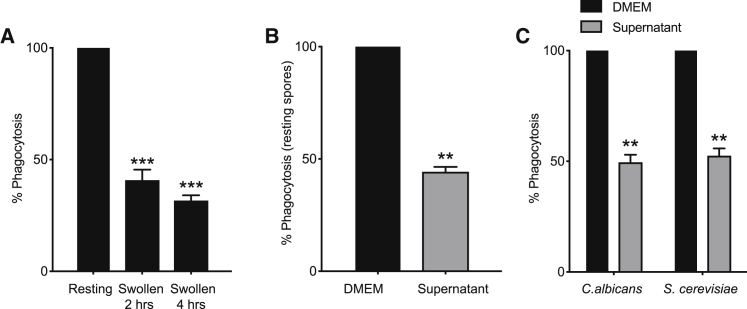
Swelling of *R. microsporus* FP469-12 spores inhibits phagocytosis (A) Phagocytosis of resting spores, or those allowed to swell for 2 or 4 h, by J447.2 macrophages. (B) Effect of swollen spore supernatant on phagocytic uptake of naive, resting *R. microsporus* spores by macrophages. (C) Effect of *R. microsporus* FP469-12 conditioned medium (Dulbecco’s modified eagle medium [DMEM]) on phagocytosis of *C. albicans* and *S. cerevisiae*. For all assays, the number of macrophages containing at least one spore were counted after 1 h. Counts were normalized to uptake of untreated resting spores in each replicate. n = 3 biological replicates of >1,000 macrophages each, error bars represent SEM. ^∗^p < 0.05, ^∗∗^p < 0.001, ^∗∗∗^p < 0.0001, one-way ANOVA with Tukey’s correction for multiple comparisons. See also Figure S1.

During these critical early stages of infection, *R. microsporus* sporangiospores become metabolically active and start to swell, prior to germination.^50^^,^^51^ This swelling is normally associated with exposure of surface MAMPs which should facilitate phagocytosis.^1^ Contrary to this expectation however, while dormant *Rhizopus* spores were readily engulfed by J774A.1 cells, swollen spores were taken up significantly less (Figure 1A, p > 0.0001). This was dependent on fungal viability as UV-killing of swollen spores completely restored their uptake (Figure S1A). Larger objects are harder for phagocytes to engulf, and under these conditions swollen spores reached a mean diameter of 7.3 μm after 6 h, compared with 4.6 μm during dormancy. However, as J774A.1 cells engulfed latex beads up to 11.9 μm in diameter at least as well as dormant spores (Figure S1B), size was not a limiting factor.

These findings suggest a novel active mechanism for evading phagocytosis upon spore germination. As fungi are well known for the production of bioactive secondary metabolites, we hypothesized a secreted factor might be responsible. We therefore allowed *Rhizopus* spores to swell in macrophage medium (Dulbecco’s modified eagle medium [DMEM]) for 1 h before removing the spores and testing the capacity of the conditioned medium supernatant to inhibit phagocytosis of other particles. This conditioned medium was sufficient to inhibit phagocytosis of dormant spores to a similar extent as swollen spores themselves (Figure 1B). Conditioned media also had a cross-protective effect on non-mucormycetes, inhibiting phagocytosis of the ascomycete yeasts *Candida albicans* and *Saccharomyces cerevisiae* (Figure 1C). *R. microsporus* FP469-12 therefore produces a secreted factor (or factors), induced upon spore swelling and metabolic activation, with broad anti-phagocytic activity.

### *R. microsporus* FP469 harbors a bacterial endosymbiont

Soil-dwelling fungi are associated with bacterial endosymbionts that modulate their host and environments through the synthesis of secreted metabolites.^36^ The plant pathogenic bacterium *Ralstonia solanacearum* produces a lipopeptide ralsolamycin that induces fungal chlamydospore formation and enables bacterial invasion of fungal cells.^38^^,^^39^ In particular, *R. microsporus* isolates can host a endosymbionts that impact plant pathogenesis: *R. microsporus* var. *microsporus* CBS 699.68 pathogenesis in rice seedlings is augmented by the endosymbiont *Mycetohabitans rhizoxinica* producing the secondary metabolite rhizoxin, a toxin that targets a conserved residue in β-tubulin, potently depolymerizing microtubules.^42^^,^^47^^,^^52^, ^53^, ^54^, ^55^ However, no involvement for endosymbionts in mammalian disease has been proven, either as a requirement for pathogenesis in humans or associated with disease severity in diabetic mice.^40^^,^^48^

**Figure 2:**
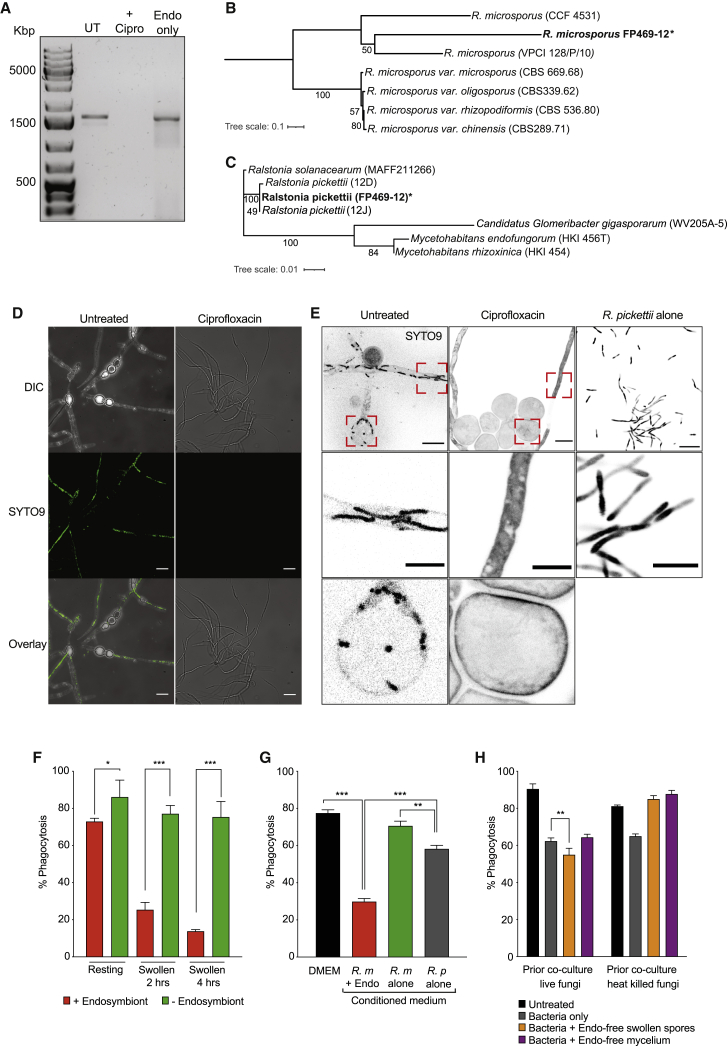
*R. microsporus* FP469-12 contains a bacterial endosymbiont required for anti-phagocytic activity (A) PCR screen for the presence of bacterial 16S rDNA. Genomic DNA was isolated from wild-type and ciprofloxacin-treated *R. microsporus* FP469-12 cells, as well as the isolated endosymbiont alone. Presence of 16S rDNA is indicated by the presence of a 1.5-kb PCR product. (B and C) (B) Phylogenetic comparison of *R. microsporus* FP469-12 and (C) its *R. pickettii* endosymbiont based on 28S and 16S sequences respectively. Both were aligned with MUSCLE, bootstrapped and produced with RAxML. Strain *R. mircosporus* var. *microsporus* is CBS 699.68, shown to harbor *M. rhizoxinica*. Strains CCF4531 and VPCI are clinical isolates from a nasal and pulmonary mass respectively. (D) SYTO9 staining of *R. microsporus* FP469-12 mycelium for bacterial endosymbionts. Spores of parent and ciprofloxacin-treated cells were fermented in VK medium, the mycelial pellet submerged in NaCl and then stained with SYTO9 prior to brightfield and fluorescence imaging. (E) High resolution confocal imaging of endosymbionts in both spores and hyphae allowed to germinate for 4 h then stained with SYTO9. *R. pickettii* alone was grown for 16 h, then stained with SYTO9. Top row shows the parental and ciprofloxacin-treated cells, and isolated *R. pickettii*, scale bars represent 10 μm. The boxed regions are enlarged below showing both hyphae (middle row) and spores (bottom) row, for each fungal strain (scale bars, 5 μm). (F) Phagocytosis of parental, and cirprofloxacin-teated (endosymbiont-free) *R. microsporus* FP469-12 spores by J774.2 macrophages, upon swelling. (G) Contributions of the fungi and bacteria to the secreted anti-phagocytic activity. J774.2 cells were incubated for 1 h with resting spores in medium conditioned by either parental spores (with endosymbionts), endosymbiont-free spores, or the isolated *R. pickettii* endosymbiont alone. (H) Effect of media conditioned by co-cultures of bacterial symbionts and endosymbiont-free fungal spores grown on phagocytosis of *R. microsporus* resting spores. Each graph shows the mean and SEM of 3 independent experiments. ^∗^p < 0.05, ^∗∗^p < 0.001, ^∗∗∗^p < 0.000, one-way ANOVA with Tukey’s correction for multiple comparisons. See also Figure S2.

To test whether an endosymbiont might influence phagocytosis, we tested for the presence of bacterial endosymbiont 16S rDNA in our *Rhizopus* strain (*R. microsporus* FP469-12), by PCR (Figure 2A). *R. microsporus* FP469-12 was positive for 16S rDNA (lane 1), which was lost upon treatment of the fungus with the antibiotic ciprofloxacin (lane 2). Through enzymatic and physical disruption of the fungal cell wall, we were able to isolate endosymbionts from FP469-12, and 16S rDNA could be amplified from the isolated bacteria, which we term RpFP469 (lane 3). For the remainder of this work, unless otherwise stated, spores treated with ciprofloxacin were germinated, passaged through sporulation twice, and frozen down for stocks before use as endosymbiont-free spores to limit the possibility that ciprofloxacin was affecting outcomes.

To further characterize the endosymbiont isolated from *R. microsporus* FP469-12, it was subjected to whole-genome sequencing. This identified the beta-proteobacterium *Ralstonia pickettii*, a relative of *Ralstonia solanacearum* and *Mycetohabitans rhizoxinica* commonly found in soil and water and occasionally associated with contaminated medical equipment as an opportunistic pathogen.^56^, ^57^, ^58^, ^59^ This was confirmed by two independent replicates identifying 16S sequences consistent with *R. pickettii* in FP469-12- but not FP469-12-cured genomic extracts (Figure 2A). *R. pickettii* has not been previously reported as an endosymbiont of *R. microsporus*. Compared with an *R. microsporus* var. *microsporus* soil isolate (CBS 699.68) known to host *M. rhizoxinica*, 28S sequence positioned FP469-12 as more similar to other *R. microsporus* clinical isolates (Figure 2B).^42^ A phylogeny based on 16S sequences placed *R. pickettii* isolated from FP469-12 as most similar to environmental isolates of *R. pickettii*, and more similar to *R. solanacearum* than to *M. rhizoxinia* or *M. endofungorum*, all known endosymbionts of *R. microsporus* (Figure 2C).

To further validate the presence and clearance of the endosymbiont, we stained *R. microsporus* FP469-12 spores with the nucleic acid-binding dye SYTO9 commonly used to stain endosymbionts within fungi.^37^ Endosymbionts were clearly visible in hyphae of the parental FP469-12 strain, but absent after ciprofloxacin treatment (Figure 2D). Higher-magnification confocal imaging enabled endosymbionts to be seen more clearly and confirmed they were intracellular in both spores and hyphae (Figure 2E). Importantly, SYTO9 staining was again lost upon ciprofloxacin treatment. Additionally, we were able to independently culture the bacterium using established growth protocols for *R. pickettii* (Figure 2E).

### Endosymbiotic *R. pickettii* are required for anti-phagocytic activity

We examined whether the presence of the bacterial endosymbiont was necessary for inhibition of fungal spore uptake by macrophages. Uptake of cured swollen spores of *R. microsporus* FP469-12 was significantly higher than that of the uncured parent (p < 0.0001) (Figure 2F). Media conditioned by endosymbiont-free spores also lost the ability to inhibit phagocytosis (Figure 2G). In addition, while the *R. pickettii* endosymbiont grew well in the absence of the fungus, it only caused a small decrease in phagocytosis when used to condition macrophage medium, although still significantly more than medium conditioned by cured spores (p < 0.001, Figure 2G). Endosymbiotic bacteria are therefore required for the secreted anti-phagocytic activity.

To further test whether the presence of intracellular bacteria was required for the anti-phagocyte activity of *R. microsporus* FP469-12, we reconstituted the endosymbiosis by co-culturing protoplasted, cured *R. microsporus* spores with the isolated *R. pickettii*. Re-establishment of the endosymbiosis was confirmed by both PCR and SYTO9 staining (Figures S2A and S2B). This fully restored the ability of swollen spores to inhibit uptake by macrophages (Figure S2C), as well as the inhibition of phagocytosis by conditioned medium (Figure S2D) and ability of the spores to evade killing by macrophages (Figure S2E).

We next assessed the individual contributions of bacteria and fungus to inhibiting phagocytosis of resting cured fungal spores (Figure 2G). There was a small but not significant decrease in phagocytosis when the conditioned media from the fungus alone was used. Conditioned media from bacteria alone caused a decrease in phagocytosis relative to conditioned media from the fungus alone although not as much as when endosymbiotic (Figure 2G). We therefore investigated whether the activities were synergistic (Figure 2H). Conditioned media generated by co-culturing bacteria with endosymbiont-free swollen spores caused a minor but significant (p = 0.019) augmentation of the inhibitory effect of *R. pickettii-*conditioned medium (Figure 2H). This effect was not present when either fungal mycelium, or heat-killed spores were used for conditioned medium (Figure 2H). While this indicates that both organisms cause a minor additive effect when cultured independently, it never reached the extent seen when growing as a true endosymbiosis. We therefore conclude that the holobiont formed by fungal/bacterial endosymbiosis is required for effective anti-phagocyte activity.

### The *Rhizopus*/*Ralstonia* holobiont blocks growth of predatory amoebas

While Mucorales can cause opportunistic infections in susceptible humans, they normally live in environments such as soil.^60^ The emergence of virulence is consequently driven by environmental interactions, rather than those between fungi and the human immune system. It has thus been proposed that mechanisms allowing evasion of phagocytic immune cells originally evolved to help fungi escape professional phagocytes in the environment, such as predatory amoebas.^3^ To test whether *R. microsporus* FP469-12 can also inhibit capture by environmental phagocytes, we examined its interactions with the soil amoeba *Dictyostelium discoideum*, a well-established model host for both pathogenic bacteria and fungi.^61^, ^62^, ^63^, ^64^, ^65^

**Figure 3:**
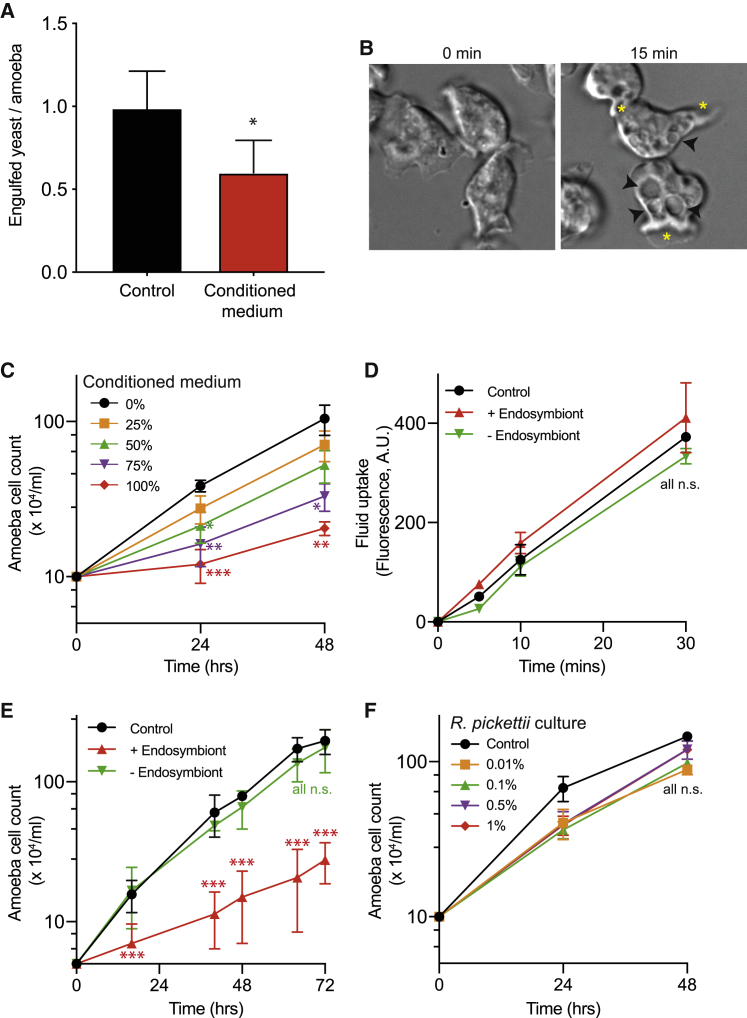
Fungal-bacterial endosymbiosis inhibits amoeba phagocytosis and growth (A) Phagocytosis of heat-killed *S. cerevisiae* by *D. discoideum* in either normal medium or medium pre-conditioned by *R. microsporus* FP469-12. (B) DIC images of *D. discoideum* cells 0 and 15 min after addition of conditioned medium. Black arrowheads indicate the large swollen vacuoles induced, yellow asterisks mark forming protrusions. (C) Dose-dependent inhibition of *D. discoideum* growth by *R. microsporus* FP469-12 conditioned medium. Amoebas were incubated in different concentrations of conditioned medium diluted in fresh medium as indicated, and cells counted at each time point. (D) Fluid uptake (macropinocytosis) by *D. discoideum* cells in medium conditioned by either parental, or endosymbiont-free *R. microsporus* FP469-12 spores. Cells were incubated in TRITC-dextran containing medium, and fluorescent dye uptake measured by flow cytometry. (E) Effect of endosymbiont removal on the ability of *R. microsporus* FP469-12 conditioned medium to inhibit *D. discoideum* growth. (F) Effect of *R. pickettii*-conditioned medium on *D. discoideum* growth. HL5 medium was conditioned for 4 h by addition of the indicated dilutions of an overnight *R. pickettii* culture, before bacteria were removed. Each graph shows the mean and SEM of 3 independent experiments. ^∗^p < 0.05, ^∗∗^p < 0.01, ^∗∗∗^p < 0.005, ANOVA with Tukey’s correction for multiple comparisons. See also Figure S3.

Consistent with our macrophage data, conditioning *D. discoideum* growth medium (HL5) with swollen *R. microsporus* FP469-12 spores for 4 h reduced the phagocytosis of inert, heat-killed *S. cerevisiae* by 41%, compared with untreated medium (Figure 3A). Time-lapse microscopy after addition of conditioned medium revealed that *D. discoideum* cells were still able to form protrusions and actively migrate over this period, indicating that they remain active and viable. However, the amoebae immediately started accumulating prominent swollen vacuoles (Figure 3B; Video S1). Factors secreted by *R. microsporus* FP469-12 therefore also affect amoeba and inhibit phagocytosis from primitive phagocytes, such as amoeba, to human phagocytes.

Video S1. Holobiont-conditioned medium affects the morphology but not motile behavior of Dictyostelium amoebae, related to Figure 3B Timelapse of *D. discoideum* cells upon exposure to media conditioned by *R. microsporus* FP469-12 spores. Conditioned media was added just before the start of the movie. Images were captured by differential interference contrast (DIC) microscopy. Note the prominent vacuolization that increases as time passes.

The accumulation of swollen vacuoles indicates that *R. microsporus* FP469-12 may cause additional disruption of intracellular vesicle trafficking. Consistent with this, *R. microsporus* FP469-12 conditioned medium caused a strong, dose-dependent inhibition of *D. discoideum* growth (Figure 3C). In liquid culture, *D. discoideum* grow using macropinocytosis to take up nutrients. We therefore measured macropinocytosis by following uptake of TRITC-dextran by flow cytometry (Figure 3D). This was unaffected by *R. microsporus* FP469-12 conditioned media, ruling out defective nutrient uptake as the cause of inhibited growth. Growth inhibition was completely dependent on the presence of the endosymbiont, as neither ciprofloxacin-treated spores nor ciprofloxacin alone affected *D. discoideum* growth, and this activity could be rescued by reconstitution of the endosymbiosis (Figures 3E and S3A). HL5 medium conditioned with concentrations of the *R. pickettii* endosymbiont well exceeding that present in the endosymbiotic cultures had little effect on *D. discoideum* growth (Figure 3F). Both bacteria and fungi are therefore essential to inhibit amoeba growth.

### *R. microsporus/R. pickettii* endosymbiosis inhibits phagosomal maturation and killing

**Figure 4:**
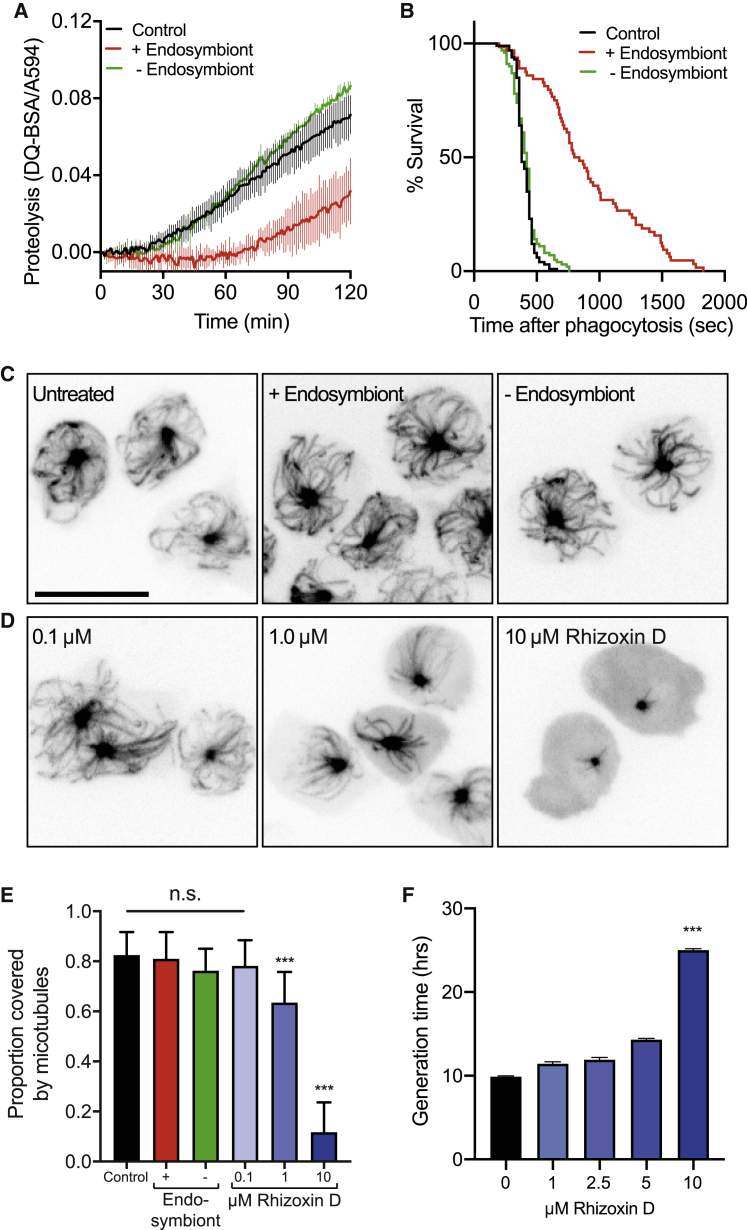
The secreted activity inhibits phagosome maturation by a novel mechanism (A) Phagosomal proteolysis of *D. discoideum* cells incubated in fungal-conditioned medium, in the presence, or absence of the endosymbiont. Measured by increasing fluorescence of DQ-BSA-conjugated beads after engulfment. (B) Kaplan-Meijer survival curve of GFP-expressing *K. pneumonia* after engulfment by *D. discoideum* in *R. microsporus* FP469-12-conditioned medium. Phagocytosis was observed by time-lapse fluorescence microscopy, and the point of bacterial death inferred from the quenching of GFP-fluorescence (n > 100 for each condition, ^∗∗∗^p < 0.001, log rank Mantel-Cox test). (C and D) (C) Maximum intensity projections of *D. discoideum* cells expressing GFP α-tubulin after 20-min treatment with either conditioned medium or (D) the indicated concentrations of rhizoxin D. (E) Quantification of the proportion of cytoplasm covered by the microtubule array in cells treated as in (C) and (D) (^∗∗∗^p < 0.001, t test). (F) Effect of rhizoxin D treatment on *D. discoideum* growth. Generation times calculated from growth curves obtained over 72 h (^∗∗∗^p < 0.001, paired t test). Unless otherwise indicated all graphs show the mean and standard deviations of 3 independent experiments.

To grow, amoebas need to both capture and digest food captured within phagosomes or macropinosomes.^66^ Newly formed phagosomes therefore represent a major interface between amoebae and ingested microbes and are a common site for evolutionary adaptations that help drive virulence. We therefore tested whether *R. microsporus* FP469-12 affected *D. discoideum* phagosomal maturation. Using DQ-BSA coated beads, which increase fluorescence upon proteolysis, we found addition of spore-conditioned medium after phagocytosis severely inhibited degradation (Figure 4A); this was again dependent on the presence of the endosymbiont. Pre-treating cells for 30 min prior to phagocytosis had no additional effect, indicating that inhibition of proteolysis was due to direct interference with the proteolytic machinery, rather than initial lysosomal delivery to the phagosome (Figure S3B).

We also measured whether *R. microsporus* FP469-12-conditioned medium inhibited the ability of amoebas to kill phagocytosed bacteria. Following the phagocytosis of non-pathogenic *Klebsiella pneumoniae* expressing GFP by time-lapse microscopy allows their intracellular survival to be measured, as the GFP-fluorescence becomes quenched upon death and permeabilization in acidic phagosomes.^67^ In untreated medium, *D. discoideum* killed 50% of engulfed bacteria within 400 s. Bacterial survival was significantly promoted by conditioned medium, and this was dependent on the presence of the endosymbiont (Figure 4B). *Klebsiella* survival was again restored for conditioned medium from spores where the endosymbiosis was reconstituted (Figure S3C). Amoeba normally feed on bacteria in the soil: *D. discoideum* were able to grow almost as fast on isolated *R. pickettii* RpFP469 as on *K. aerogenes* bacteria widely used in laboratory culture (Figure S3D). We therefore propose predation constitutes an important evolutionary driver of endosymbiosis, selecting for collaboration between bacteria and fungi to inhibit both uptake and killing by amoeboid predators.

### Amoebas are inhibited by a novel, microtubule-independent activity

Endosymbionts of fungi commonly influence their environments via the secretion of bioactive metabolites. For example, *R. solanacearum* produces the lipopeptide ralsolamycin, which enables invasion of target fungi, via the activity of two non-ribosomal peptide synthetase (NRPS) genes.^38^^,^^39^ The *M. rhizoxinica* endosymbiont synthesizes the rhizoxin family of secondary metabolites via a *trans-*acyl transferase non-ribosomal peptide synthase polyketide synthase (*trans-*AT NRPS PKS) gene cluster.^32^^,^^55^ Rhizoxin directly binds the β-tubulin subunit of microtubules, potently causing their depolymerization and mitotic arrest in both plants and humans.^52^ It is thought that this endosymbiosis emerged to help *R. microsporus* damage and saprophytically feed on plant tissue.^68^ Loci for both ralsolamycin and rhizoxin derivatives are widespread: we identified sequences consistent with the *rhi* locus responsible for rhizoxin biosynthesis in *R. solanacearum* genomes, and the analysis of *M. rhizoxinica* genome HKI 454 identified a locus with 40% similarity to ralsolamycin.^69^ We therefore asked whether *R. pickettii* might similarly encode biosynthesis of ralsolamycin or rhizoxin derivatives or other known metabolites. We performed an unbiased genomic analysis of the *R. pickettii* isolated from *R. microsporus* FP469-12, as well as publicly available *R. pickettii* genomes from environmental sources. AntiSMASH-6 software for secondary metabolite clusters revealed similar overall architecture across the three isolates, including biosynthetic pathways for siderophores, bacteriocins, terpenes, and arylpolyenes.^70^ Uniquely, *R. pickettii* RpFP469 encodes a type I polyketide synthase (TIPKS) lacking similarity to known T1PKS, and this locus was specifically expressed under conditions that induced expression of the anti-phagocytic factor (Figure S4A). Blast analysis identified similar loci in *R. pickettii* (FDAARGOS_1535 and K-288, 84%) and *R. insidiosa* (FC1138, 79%) genomes, but not in *R. solanacearum* or *M. rhizoxinica* genomes. Loci consistent with either ralsolamycin or rhizoxin were not identified in any of the tested *R. picketti* genomes.

The microtubule cytoskeleton is important for many cellular functions, including vesicle trafficking, which is important for phagosome maturation. Therefore, we investigated whether microtubules might be a target of the *R. microsporus-R. pickettii* endosymbiosis. Contrary to this hypothesis, treatment of *D. discoideum* cells expressing GFP-α-tubulin with *R. microsporus* FP469-12 conditioned medium caused no measurable depolymerization of the microtubule cytoskeleton either with or without the endosymbiont (Figure 4C). This was quantified by the extent of the microtubule array as a proportion of the cell area (Figure 4E). As a positive control, we also treated the cells with rhizoxin D. This was highly effective at depolymerizing microtubules, but only at concentrations above 1 μM (Figures 4D and 4E). This is 5 orders of magnitude higher than that required for a similar effect in mammalian cells,^47^ even though all residues at the β-tubulin rhizoxin-binding site are conserved.^52^ Furthermore, while conditioned medium blocks *D. discoideum* growth without obvious microtubule disruption, rhizoxin D only inhibited growth at concentrations where microtubules were almost completely depolymerized (Figure 4F). We therefore conclude that the *R. icrospores-R. pickettii* endosymbiosis inhibits amoeba function via a novel, most likely microtubule-independent, mechanism.

### Endosymbiosis facilitates fungal evasion of macrophages

**Figure 5:**
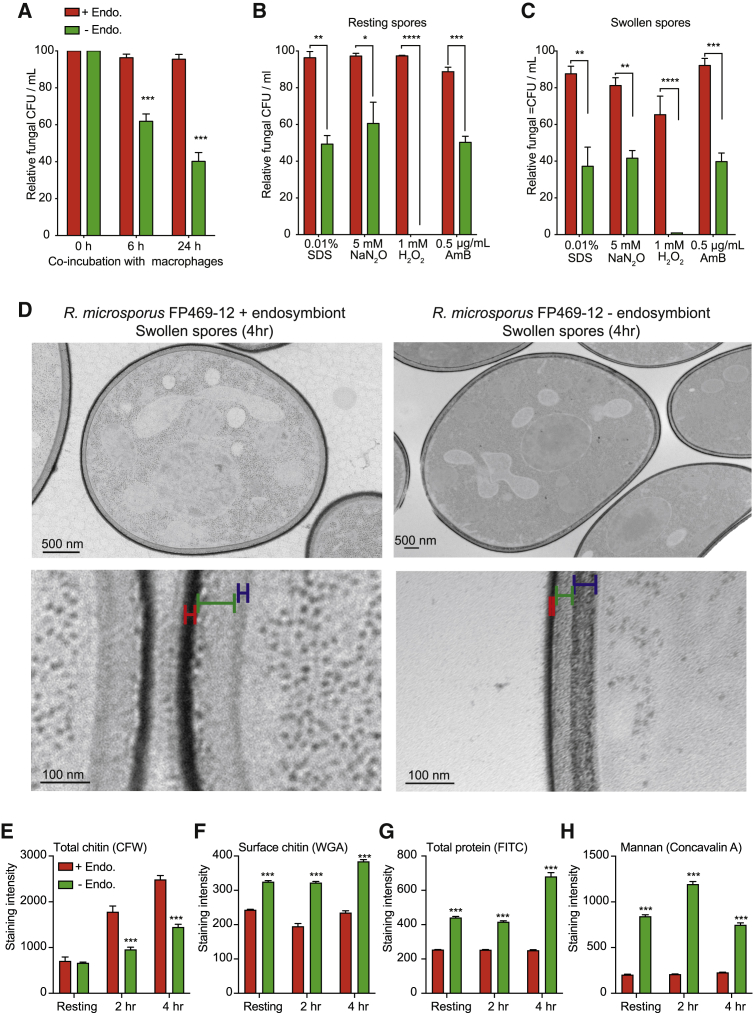
Endosymbiosis with *R. pickettii* influences the fungal cell wall and stress tolerance (A) Survival (CFUs) of *R. microsporus* FP469-12 resting spores after incubation with J774.1 macrophages in the presence and absence of endosymbiont. (B and C) Impact of the endosymbiont on *R. microsporus* FP469-12 spore survival under cell wall, nitrosative, oxidative, and antifungal stresses (AmB: Amphotericin B). Either resting (B) or swollen (C) spores were incubated at the indicated concentrations for 24 h, prior to CFU determination. (D) TEM images of spores swollen for 4 h in the presence or absence of the endosymbiont. Lower panels show enlargements of representative cell wall regions. Corresponding regions with comparable density are indicated by the different colored bars. (E–H) Show comparative changes in cell wall composition upon swelling of the parental FP469-12 strain (+Endo) and endosymbiont-free fungal spores (−Endo). Staining intensities were quantified by fluorescence microscopy. (E) shows total chitin (calcofluor white), (F) exposed chitin (wheat-germ agglutinin), (G) total protein (FITC), and (H) mannan (concanavalin A) (n = 300 for each repeat). All graphs show mean ± SEM of 3 repeats (^∗∗^p < 0.001, ^∗∗∗^p = 0.0001, ^∗∗∗∗^p < 0.00001, one-way ANOVA with Tukey’s correction for multiple comparisons). See also Figure S4.

The data above show that endosymbiosis allows *R. pickettii* and *R. microsporus* FP469-12 to inhibit both phagocytosis and phagosomal killing by amoebae. To test whether this promotes virulence in animals, we first tested how the presence of the endosymbiont effected evasion from J774A.1 macrophages. Co-incubation showed that spores containing endosymbiont were highly resistant to clearance by macrophages, with no significant decrease in colony forming units (CFUs) over 24 h. In contrast, spores lacking endosymbionts were cleared much more effectively, with 60% removed within 24 h (Figure 5A, p < 0.0001).

The secreted activity identified above may not be the sole protective mechanism conferred by the endosymbiont. Previous work has shown that endosymbiosis with *M. rhizoxinica* is also beneficial for fungal development, cell wall synthesis, and stress resistance.^43^^,^^44^^,^^71^ As the fungal cell wall is the main interface between the engulfed fungi and the antimicrobial activities of the phagosome, we investigated whether *R. pickettii* endosymbiosis also affects *R. microsporus* resistance to phagosome-relevant stresses. In the absence of the endosymbiont, both resting and swollen spores were significantly more sensitive to treatment with 0.01% sodium dodecyl sulphate (SDS), 5-mM NaNO_3_, or 1-mM H_2_O_2_, as well the front-line antifungal amphotericin B (AmB, 0.5 μg/mL) (Figures 5B and 5C). Endosymbiosis with *R. pickettii* therefore increases the resistance of *R. microsporus* to several physiologically important antimicrobial stresses.

As activation of phagocytic receptors by fungal surface ligands is critical for engulfment, we measured how the fungal cell wall changed upon swelling and how this was influenced by the presence of the endosymbionts. Transmission electron microscopy (TEM) of swollen spores with and without the endosymbiont revealed differences in electron density and width of the cell wall interior, suggesting that the endosymbiont influences spore cell wall organization (Figure 5D).

To further characterize the impact of the endosymbiont, fungi were stained and imaged to reveal specific cell wall components. This showed that in the parental FP469-12 strain, total chitin (stained with calcofluor white [CFW]) increased more than 2-fold upon spore swelling (Figure 5E), although surface exposure of chitin (WGA, wheat-germ agglutinin) and total protein (FITC, fluorescein isothiocynate) were unaltered between resting and swollen spores (Figures 5F and 5G). We were unable to detect any β-glucan exposure, consistent with previous reports showing low glucan in *Rhizopus* species spore cell walls.^4^^,^^72^^,^^73^ Instead, mannan (stained with the lectin concavalin A) was detected as a major component of the cell wall at all stages of growth: exposed mannan was detected on the surface of all cells and was particularly high in approximate half of resting spores and swollen (Figures 5H and S4B).

When the endosymbiont was removed, polysaccharide levels still increased, although there was less CFW staining at each time point compared with the parental strain (Figure 5E). In contrast to the parental strain, spores lacking endosymbionts had higher levels of exposed chitin (WGA), total protein (FITC), and mannan levels at all time points (Figures 5G and 5H). Both surface chitin and total protein increased further after 4 h swelling, with a transient increase in mannan (Con A, concanavalin A) at 2 h swelling (Figure 5H, p < 0.0001). Consistent with this broad impact on fungal cell wall organization and stress resistance, we observed a decrease in spore production when the cleared strain was serially passaged (Figure S4C). The presence of the endosymbiont therefore has broad influence on the structure of the cell wall during swelling and protects both the fungus and bacteria from environmental phagocytes and immune cells by multiple mechanisms.

### Endosymbiosis with *R. pickettii* is required for immune evasion and virulence *in vivo*

**Figure 6:**
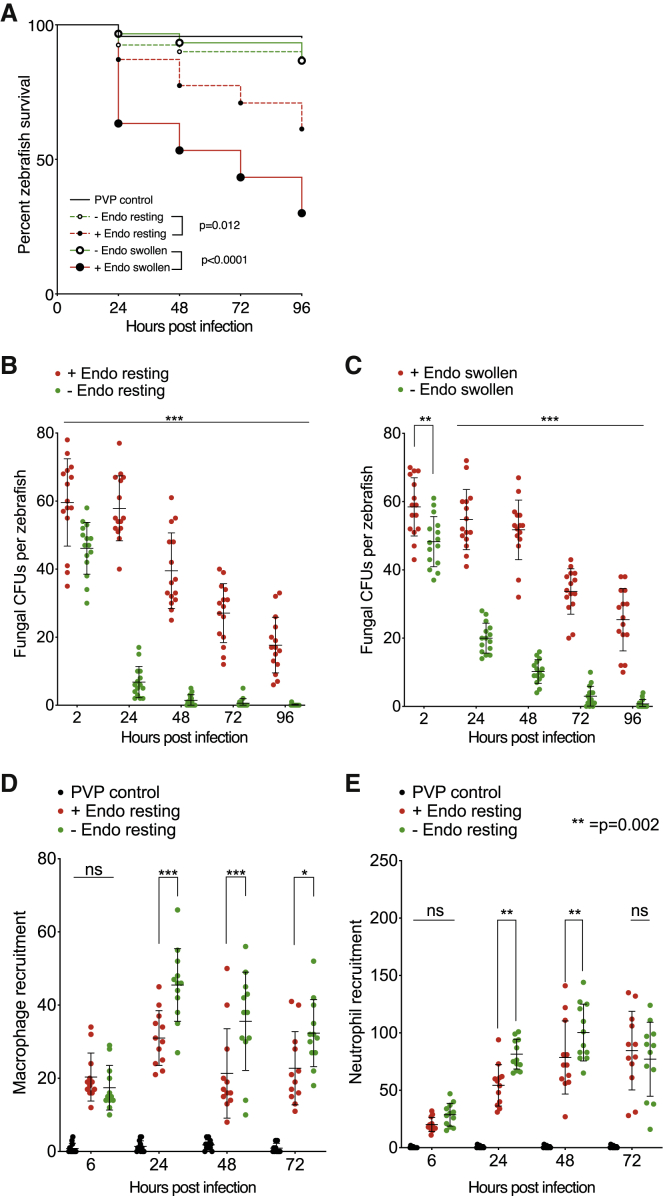
Effect of endosymbiont status on fungal infection of zebrafish (A) Survival of AB wild-type zebrafish injected via the hindbrain with resting or swollen spores of *R. microsporus* FP469-12 in the presence or absence of endosymbiotic bacteria. PVP indicates mock-injected fish. Three biological replicates of populations of 10 fish each were examined (n = 30). Statistical differences were determined using Mantel-Cox with Bonferroni’s correction for multiple comparisons (5% family-wise significance threshold = 0.025). (B and C) Effect of endosymbiont status on fungal survival (CFUs) following hindbrain injections of AB wild-type zebrafish with (B) resting or (C) swollen *R. microsporus* FP469-12 spores. Three biological replicates of 5 fish per condition were examined (n = 15). (D and E) Effect of the endosymbiont on *in vivo* recruitment of macrophages and neutrophils to the site of infection. Figure S5 shows representative images and Figure S6 shows equivalent data with swollen spores. Statistical significance was assessed by two-way ANOVA with Tukey’s correction for multiple comparisons or pairwise t tests where sample number was unequal due to fish death, ^∗^p < 0.05; ^∗∗^p < 0.001; ^∗∗∗^p < 0.0001 unless otherwise indicated.

Finally, we investigated the influence of bacterial-fungal symbiosis in Mucorales pathogenesis *in vivo*. Using our recently established zebrafish (*Danio rerio*) model of infection,^49^ wild-type larvae were infected with either resting or swollen wild-type or cured spores (Figure S5A). Over 96-h post-infection (hpi), *R. microsporus* FP469-12 spores containing the endosymbiont killed a significant proportion of the larvae, with swollen spores being more virulent than resting (65% mortality at 96 hpi, compared with 40%, Figure 6A). In contrast, spores lacking the endosymbiont were completely avirulent, with neither swollen nor resting spores statistically different from mock injection in this model (p > 0.05). This correlated with CFUs over time: while there were no differences in initial inocula, fungal CFUs from fish infected with endosymbiont-free spores were significantly reduced within just 2 h and continued to be more rapidly cleared than wild-type spores over 96 h (Figure 6B). This was independent of whether fish were infected with resting or pre-swollen spores, although in both cases, resting spores were more rapidly cleared (Figure 6C). Together, these data suggest that the endosymbiont aids immune evasion during the initial phase of infection and that this is exacerbated by metabolic pre-activation of spores.

Disease outcome correlates with the peak number of phagocytes at the site of infection (carrying capacity) in the first 24 h, and successful infection control requires spore killing.^18^^,^^49^ To investigate the role of the endosymbiont in modulating host defense, we quantified immune cell recruitment using transgenic zebrafish where either macrophages or neutrophils were fluorescently labeled (Tg(mpeg1:G/U:NfsB-mCherry or Tg(mpx:GFP)^i114^, respectively^74^^,^^75^) (Figure S5). Zebrafish were therefore infected with wild-type or endosymbiont-free resting spores and the number of phagocytes recruited to the site of infection counted over the following 96 h (for survival curves, see Figures S6A and S6B).

24 hpi, both macrophage and neutrophil recruitment was significantly lower in larvae injected with spores containing the endosymbiont than those without (Figures 6D and 6E, p < 0.0001). This difference was sustained over 48 h but became less significant at 72 hpi, most likely due to different disease progression as endosymbiont-free spores are cleared (Figure 6A). Similar results were observed with spores pre-swollen before injection (Figures S6C and S6D). While fish that failed to recruit macrophages after 24 h did not survive until the end of the assay regardless of endosymbiont status, only macrophages recruited to endosymbiont-free infections efficiently contained the infection. While it remains an open question whether recruitment is inhibited by secreted factors, or other changes in fungal biology mediated by the endosymbiont, the presence of *R. pickettii* enhances *R. microsporus* virulence *in vivo* by multiple mechanisms, leading to suppression of the immune response and fungal clearance.

**Figure 7:**
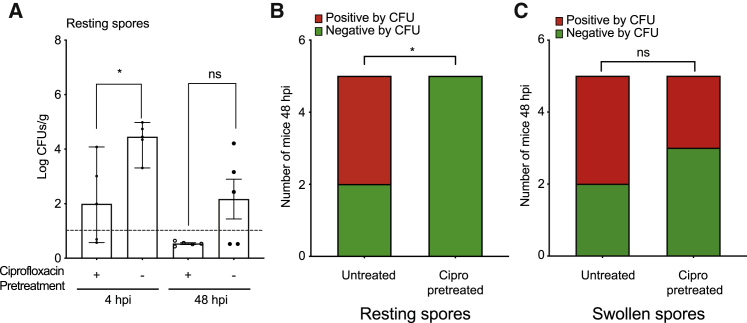
Ciprofloxacin treatment can modify *R. microsporus* FP469-12 infection in mice. (A) Fungal survival (CFUs) at 4 and 48 following intra-tracheal infection of mice hours with resting spores. Where indicated, spores were pre-treated with 60 μg/mL ciprofloxacin for 3 h prior to infection. (n = 5, significance determined by Mann-Whitney test.) (B and C) Proportion of mice positive or negative (below the detection limit) for fungal CFU’s 48 hpi. (B) Data from infection with resting spores with and without ciprofloxacin pre-treatment, (C) is the same experiment performed with swollen spores. n = 5 mice per condition, statistical significance assessed using a two-sided chi-squared test, and attributable risk was assessed using Newcombe/Wilson with continuity correction, ^∗^p = 0.384.

As endosymbiont removal promoted spore clearance in zebrafish, we attempted to model the impact of antibiotic treatment immediately prior to or during infection on disease outcome. Concomitant treatment of the fish water with 60 μg/mL ciprofloxacin had no effect on fish survival or spore CFU upon infection with either resting or swollen spores (Figures S6E and S6F). Unfortunately, it is not possible to differentiate whether this is due to lack of effect or insufficient drug permeability. Therefore, we instead performed a small proof of concept pilot study in immune-competent mice (n = 5 per group). Mice were infected intra-tracheally with resting spores that had been untreated or treated with ciprofloxacin for 3 h immediately prior to inoculation and CFUs measured after 4 and 48 h. Similar to the rapid spore killing observed in fish (Figure 6B), within 4 h, there was a reduction in CFUs recovered from mouse lungs infected with ciprofloxacin-treated resting spores (p = 0.019) (Figure 7A). We ruled out a direct inhibitory effect of ciprofloxacin on fungal survival as swollen spores treated with ciprofloxacin were as resistant to host killing as untreated swollen spores (p = 0.095) (Figure 7C). After 48 h, while 3/5 mice infected with untreated resting spores remained positive by CFU, all 5 mice infected with ciprofloxacin-treated resting spores cleared the infection (Figure 7B). While underpowered, this was statistically significant (p = 0.0384, chi-square test, Newcombe/Wilson with continuity correction, with 60% attributable risk). This effect was lost when mice were infected with pre-swollen spores, where any secreted factors are washed away prior to infection (Figure 7C). Therefore, while endosymbiosis most likely evolved to promote survival in the environment, it also confers protection from phagocytic immune cells in animals, demonstrating an important role in facilitating opportunistic infection.

## Discussion

In this work, we describe an endosymbiosis between the opportunistic fungal pathogen *Rhizopus microsporus* and the gram-negative bacterium *Ralstonia pickettii*. Both are widely distributed in the environment, normally living in the soil where they will be preyed upon by microbial phagocytes, such as amoebas. A wide diversity of fungi harbor bacterial endosymbionts that influence fungal phenotypes related to metabolism, cell wall organization, development, and plant host colonization.^40^^,^^42^^,^^43^^,^^45^^,^^47^^,^^54^^,^^76^, ^77^, ^78^, ^79^, ^80^, ^81^, ^82^, ^83^, ^84^, ^85^, ^86^, ^87^ Endobacteria are also a source of potent mycotoxins that can influence fungal pathogenesis in plants and insects.^42^^,^^46^^,^^47^^,^^54^^,^^88^^,^^89^ Here, we demonstrate a novel interaction, whereby endosymbiosis between *R. microsporus* and *R. pickettii* enables the biosynthesis and secretion of factors that inhibit both the growth of amoebas, and their ability to capture and kill other microbes. We propose that the tri-kingdom interaction with amoebas provides an additional evolutionary driver for endosymbiosis, enabling both bacteria and fungus to evade predation in the environment.

While not all isolates of *R. microsporus* (*Rm*) harbor endosymbionts, there are examples of *Rm*-*Mycetohabitans* endosymbioses that are essential for asexual sporangiospore and sexual zygospore production.^43^^,^^45^ In these partnerships, endobacteria reside within the fungal cytoplasm and are vertically transmitted through sporulation and mating.^43^^,^^45^ Here, we similarly observe vertical transmission of *R. pickettii* in sporangiospores, with multiple bacteria apparent per spore, and observed a reduction in sporangiospore production after repeated passage of cured fungi, suggesting large scale regulatory changes as a result of endosymbiont loss. Consistent with mutualism, the presence of the *R. pickettii* endosymbiont caused widespread changes to its host; endosymbiont removal altered *R. microsporus* cell wall structure and sporangiospore viability and reduced resistance to reactive nitrogen and oxygen species as well as engulfment and phagocyte-mediated killing. Loss of the endosymbiont also increased sensitivity to the front-line antifungal treatment amphotercin B. This indicates altered ergosterol content and suggests an additional way in which the endosymbiont may influence the outcome of human infections.

Endosymbionts of fungi can employ multiple strategies to modulate their hosts and environment. Our analysis of the *R. pickettii* genome did not identify loci consistent with known metabolites ralsolamycin or rhizoxin but did identify a unique T1PKS that was specifically expressed during interaction with the fungus. Endosymbiosis-dependent expression of biosynthetic gene clusters (BGCs) has been observed in *R. microsporus-M. rhizoxinica* interactions, including for conserved factors that support invasion and endosymbiosis.^90^, ^91^, ^92^ However, the *R. pickettii* locus lacked similarity to known metabolites and not present in the closely related environmental *R. pickettii* isolates 12D and 12J.^32^^,^^39^^,^^55^ The locus does share 79% similarity with the emerging pathogen *R. insidiosa*.^93^ Interestingly, comparison to the MIBiG library of BGCs did not identify similarity to secondary metabolites of bacterial species, but rather identified metabolites associated with a variety of soil fungi.^94^ There was no evidence that this locus shared similarity with *Rhizopus* species genomes, excluding horizontal gene transfer of the locus from the host fungus. Future work will further investigate the nature of this product.

Most studies of *R. microsporus* secreted mycotoxins have focused on molecules generated by *M. rhizoxinica* and related endosymbionts of the *Burkholderia* genus.^46^, ^47^, ^48^^,^^54^^,^^82^^,^^90^^,^^95^ Although *Ralstonia*, *Burkholderia*, and *Mycetohabitans* spp. are related and share the same niche, the anti-amoebic activity of the *R. microsporus* FP469-12 endosymbiosis differs in several important ways. First, while *M. rhizoxinica* alone is sufficient for rhizoxin-derivative biosynthesis,^47^^,^^55^ media conditioned with the isolated *R. pickettii* endosymbiont from *R. microsporus* FP469-12 was ineffective against either *D. discoideum* or macrophages, indicating in this case both fungi and bacteria are necessary for the secreted bioactivity. The mechanism of action also differs compared with rhizoxin; while rhizoxin potently depolymerizes the microtubule cytoskeleton, this appeared unaffected in amoebas treated with *R. microsporus* FP469-12-condiditioned medium. Interestingly, while rhizoxin is effective at picomolar concentrations in plants and mammalian cells,^47^^,^^53^ *D. discoideum* were several orders of magnitude more resistant to rhizoxin D. This is most likely due to the highly active multi-drug efflux pumps in amoebas that presumably evolved for precisely this reason—to protect against environmental toxins. While we cannot exclude more subtle effects on, for example, microtubule motors, almost complete depolymerization of the microtubule array was required to inhibit growth to a similar extent as conditioned medium. Future studies are needed to identify the active molecule and target, but it seems likely that amoebas growth and phagosome maturation are perturbed by a novel mechanism.

An interesting additional aspect to the relationship between environmental fungi, bacteria, and amoebas is that bacteria can also form endosymbiotic relationships with amoebas.^96^^,^^97^ In particular, several *Burkholderia* spp have been identified as stable symbionts associated with environmental isolates *D. discoideum*.^98^, ^99^, ^100^ The relationships between amoeba and symbionts are complex and not always obvious, with both costs and benefits to both organisms described.^97^^,^^100^^,^^101^ Nonetheless, the different symbiotic relationships between bacteria, fungi, and amoebae, within the same environmental niche, underlines the multifactorial interactions occurring in complex microbial communities.

As the majority of fungi live in the environment and do not normally infect animals, how they evolved the mechanisms that allow them to evade the human immune system and cause opportunistic infections is unclear. As phagocytosis and phagosome maturation are highly conserved across evolution,^102^ it has been proposed that opportunistic fungal virulence derives from environmental interactions with amoebas.^3^^,^^103^ Our data show that the presence of the endosymbiont confers resistance to macrophages in a similar manner to amoebae, inhibiting both phagocytosis and killing. The presence of the endosymbiont is also essential for virulence in a zebrafish model of infection. While the widespread changes in fungal physiology that occur upon endosymbiont removal indicate that evading amoebas is not the sole driver of endosymbiosis, we suggest that it is an important contributary factor, and significant for the evolution of virulence.

While a broadening diversity of basal saprophytic fungi are a major emerging cause of clinically important superficial and disseminated fungal infections, the role of endosymbionts in pathogenesis has yet to be clinically defined.^11^^,^^104^ Two groups recently reported that although bacterial endosymbionts, including rhizoxin and rhizonin producers, are widely found among mucormycete clinical isolates, these endosymbionts did not impact pathogenesis during mucormycosis in both mouse and fly models.^40^^,^^48^ In a diabetic mouse model infected with *Rhizopus* isolates harboring rhizoxin-producing *Burkholderia* endosymbionts, no impact of treatment with ciprofloxacin 24 hpi was observed.^40^ In contrast, we found that pretreatment of resting spores with ciprofloxacin before infecting immune-competent mice significantly reduced CFUs retrieved after 4 h, and spores could no longer be recovered after 48 h. This benefit was however lost when spores were pre-swollen prior to infection, indicating that to be therapeutically effective, endosymbiont removal would need to occur early in the infection. While our study only used a small number of mice (5 per group) and is therefore prone to the challenges associated with underpowered models of infection, similar patterns were observed in zebrafish, with much larger sample sizes. While most studies to date have focused on *Burkholderia* spp endosymbionts and the rhizoxin and rhizonin mycotoxins, our work indicates a broader diversity of both endosymbionts and virulence mechanisms. The relevance of fungal-bacterial endosymbiosis to human infections therefore needs readdressing, using larger-scale studies of the prevalence and diversity of endosymbionts in both clinical and environmental isolates.

Our findings highlight the potential importance of bacterial symbiosis in *R. microsporus* pathogenesis. However, we urge caution in extending our findings to all Mucorales or fungal endosymbionts. While a number of studies have demonstrated general features common to host-Mucorales interactions, there are also likely to be species-specific aspects. In addition, not all clinical isolates profiled harbor endosymbionts, suggesting that they may augment, but are not constitutively required for, pathogenesis.^40^ Overall however, this work points to a new role for bacterial endosymbionts in fungal immune evasion, driven by environmental interactions with amoebae. The observation that endosymbiont elimination impacts phagocytosis, cell wall organization, stress resistance, and antifungal drug susceptibility as well as resistance to macrophage-mediated killing raises the prospect that endosymbiont-targeted treatments may be useful in the treatment and management of a subset of opportunistic fungal infections.

## STAR★Methods

### Key resources table

**Table:** 

REAGENT or RESOURCE	SOURCE	IDENTIFIER
**Antibodies**		

Dectin-1 IgG antibody	Gordon Brown lab^105^	Fc Dectin-1; RRID:AB_2904615
goat anti-mouse IgG-488	Sigma Aldrich	Cat# SAB4600387-50UL; RRID:AB_2904614

**Bacterial and Virus Strains**		

GFP-expressing *Klebsiella pneumoniae* fluorescence	Pierre Cosson, University ofGeneva.	N/A
*Ralstonia pickettii* RpFP469	Isolated from *R. microsporus* FP469-12, originating from a human infection at the Queen Elizabeth Hospital Birmingham Trauma Centre, UK, by Dr. Deborah Mortiboy	Strain ID RpFP469

**Biological Samples**		

*Rhizopus microsporus* strain FP469-12.6652333	Isolated from a human infection at the Queen Elizabeth Hospital Birmingham Trauma Centre, UK, by Dr. Deborah Mortiboy	Strain ID FP469-12.6652333
*Candida albicans*	Neil Gow, University of Exeter	Strain ID SC5314
*Saccharomyces cerevisisae*	Neil Gow, University of Exeter	Strain ID AM13/001

**Chemicals, Peptides, and Recombinant Proteins**		

HL5 medium	Formedium	Cat# HLG0102
DQ-green BSA	Invitrogen	Cat# D12050
Alexa594-NHS ester	Invitrogen	Cat# A37572
LoFlo medium	Formedium	Cat# LF0501

**Critical Commercial Assays**		

Live/Dead BacLight bacterial viability kit	ThermoFisher Scientific	Cat# L7007
DNeasy® powerlyzer® microbial kit	Qiagen	Cat# 12255-50
Phusion High Fidelity PCR kit	New England Biolabs	Cat# E0553S

**Deposited Data**		

*Ralstonia pickettii RpFP469*whole genome and RNAseq	Sequence Read Archive (SRA)	Accession # SRR16523121
*Rhizopus microsporus FP469-12* whole genome	Sequence Read Archive (SRA)	Accession # SRR16610381
*Ralstonia solanacearum*16S	GenBank	Accession # AB024604.1
*Candidatus Glomeribacter gigasporarum* 16S	GenBank	Accession # AJ251633.1
*Mycetohabitans endofungorum* 16S	GenBank	Accession # AM420302.1
*Ralstonia pickettii* 12J 16S	GenBank	Accession # CP001068.1
*Ralstonia pickettii* 12D 16S	GenBank	Accession # CP001644.1
*Mycetohabitans paraburkholderia rhizoxinica* HKI 454 16S	GenBank	Accession # FR687359.1
*Rhizopus microsporus* CCF4531 23S	GenBank	Accession # HG324080.1
*Rhizopus microsporus* VPCI 128/P/10 23S	GenBank	Accession # KJ417570.1
*Rhizopus microsporus* var. *oligosporus* 23S	GenBank	Accession # MH869766.1
*Rhizopus microsporus* var. *microsporus* 23S	GenBank	Accession # MH870925.1
*Rhizopus microsporus* var. *chinensis* 23S	GenBank	Accession # MH871901.1
*Rhizopus microsporus* var. *rhizopodiformis* 23S	GenBank	Accession # MH873059.1

**Experimental Models: Cell Lines**		

J774A.1	ATCC	Strain ID TIB-67
*Dictyostelium discoideum* Ax2 axenic strain	www.dictybase.org	Strain ID DBS0235521

**Experimental Models: Organisms/Strains**		

wild type AB zebrafish	Steve Renshaw, University of Sheffield	N/A
GFP-neutrophil zebrafish line	Steve Renshaw, University of Sheffield^74^	Genotype Tg(mpx:GFP)^i114^)
GFP-macrophage zebrafish line	Steve Renshaw, University of Sheffield^75^	Genotype Tg(mpeg1:G/U:NfsB-mCherry)
CD-1 20-23g male mice	Envigo, Indianapolis, IN, USA	Strain ID Hsd:ICR (CD-1®)

**Oligonucleotides**		

5’CCGAATTCGTCGACAACAGAGTTTGAT CCTGGCTCAG3’	^106^	Primer 16S rDNA Universal F
5’CCCGGGATCCAAGCTTACGGCTACCTT GTTACGACTT 3’	^106^	Primer 16S rDNA Universal R
5’GTCTTTCCTTCTATTGTTGGTC 3’	^40^	Primer Rm*ACT1*-F
5′-CCATCAGGAAGTTCATAAGAC-3′	^107^	Primer Rm*ACT1*-R
5’ATGATCTAGCTTGCTAGATTGAT 3’	^108^	Primer Rp-F
5’ACTGATCGTCGCCTTGGTG 3’	^108^	Primer Rp-R

**Recombinant DNA**		

*Dictyostelium* GFP-tubulin expression vector	^109^	Plasmid pJSK336

**Software and Algorithms**		

ImageJ	https://imagej.nih.gov/ij/^110^	N/A
kraken (v1)	https://ccb.jhu.edu/software/kraken/^111^	N/A
Graphpad Prism 7	Graphpad	N/A
Hisat2 (Version 2.0.5)	http://www.ccb.jhu.edu/software/hisat/index.shtml^112^	N/A
HTSeq (Version 0.10.0)	https://htseq.readthedocs.io/en/release_0.10.0/^113^	N/A
edgeR (Version 3.16.5)	https://bioconductor.org/packages/release/bioc/html/edgeR.html^114^, ^115^, ^116^	N/A
RAxML	https://github.com/stamatak/standard-RAxML^117^	N/A
MUSCLE	https://www.ebi.ac.uk/Tools/msa/muscle/^118^	N/A

### Resource availability

#### Lead contact

Further information and requests for resources and reagents should be directed to and fulfilled by the lead contact Elizabeth Ballou (E.Ballou@Exeter.ac.uk).

#### Materials availability

Strains unique to this study will be made available to other users upon request.

### Experimental model and subject details

#### *R. microsporus* strain and culture

*Rhizopus microsporus* strain FP469-12.6652333 was isolated from a human infection at the Queen Elizabeth Hospital Birmingham Trauma Centre by Dr. Deborah Mortiboy. This was routinely cultured on Sabouraud dextrose agar (SDA) (EMD Millipore co-operation) or potato dextrose agar (PDA) to induce sporulation at room temperature. Sporangiospores were collected by gentle disruption of the mycelium in the presence of PBS. Harvested spores were filter through a 70 μm filter to exclude hyphal fragments, then washed and counted by haemocytometer. Routine liquid cultivation was performed in 500 mL conical flasks containing 250 mL of serum free Dulbecco’s modified eagle’s media (with 1% penicillin/streptomycin and L-glutamine) (DMEM). Fungi grown in this way was used for metabolic activation of spores, to prepare swollen spore supernatants, and for DNA isolation.

#### *R. picketti* strain and culture

*Ralstonia pickettii* strain RpFP469 was isolated from *R. microsporus* FP469-12.6652333 spores incubated in SabDEX overnight and then macerated with sterilized glass beads. The resulting supernatant was filtered through decreasing filter sizes (70 μm to 3 μm) to remove fungal particles and plated onto LB agar to allow bacterial growth. Isolated colonies were incubated in LB liquid medium at 30 degrees, 200rpm overnight, then washed and stained with the Live/Dead BacLight bacterial viability kit (Thermofisher Scientific) according to the manufacturer’s instructions.

#### Macrophage cell line culture

J774A.1 murine macrophage-like cells were maintained in DMEM supplemented with 10% foetal bovine serum, 1% Streptomycin (100 μg/mL), penicillin (100 U/mL), and 1% L-glutamine (2 mM). The cells were cultivated in a humidified environment at 37^°^C enriched with 5% CO_2_ and used between 3 and 15 passages after thawing.

#### D. discoideum culture

All experiments were performed using the Ax2 axenic strain, routinely subcultured in HL5 medium (Formedium) at 22 ^°^C. For growth curves, cells were seeding cells at 1 x 10^5^/ml in the appropriate medium and counted at regular intervals until out of exponential growth. GFP-α tubulin was expressed using extrachromosomal vector pJSK336^109^; cells were transformed by electroporation and transformants selected in 20 μg/mL G418. Using ImageJ,^110^ maximum intensity projections of images from a Perkin-Elmer Ultraview VoX (60X 1.4NA objective) were then used to manually draw around the tips of the microtubule array, and calculate the areas covered as a proportion of the whole cytosol.

#### Zebrafish maintenance and infection

Infections were performed in wild type AB zebrafish, as well as GFP-neutrophil (Tg(mpx:GFP)^i114^) lines.^74^ Macrophage-specific mCherry expression was achieved by crossing Tg(mpeg1:Gal4-FF)^g125^ with Tg(UAS-E1b:NfsB.mCherry)^c264^, referred to as Tg(mpeg1:G/U:NfsB-mCherry).^75^ Zebrafish were cultivated under a 14 h-10 h light-dark cycle at 28°C at the University of Birmingham (BMS) Zebrafish Facility. All zebrafish care protocols and experiments were performed in accordance with the UK animals (scientific procedures) act, 1986. Following collection of the eggs, at 40-60 eggs per 25 mL E3 media (plus 0.1% methylene blue and 1 mg/mL 1-phenyl-2- thiourea (PTU)) for 24 h and larvae were incubated at 28°C. PTU at this concentration prevented melanization of the embryos. All media and reagents used was purchased from Sigma Aldrich unless otherwise mentioned.

#### Mouse husbandry

All experiments were performed using immunocompetent CD-1 20-23g male mice. Group size was determined from previous experiments as the minimum number of mice needed to detect statistical significance (P < 0.05) with 90% power. Mice were randomly assigned to groups by an investigator not involved in the analysis, and the fungal inocula were randomly allocated to groups. Mice were house in individually ventilated cages (IVCs), 5 per cage, and were provided with food and water ad libitum. Inocula were delivered in an unblinded fashion.

#### Ethical statement

All maintenance protocols and experiments were performed in accordance to Animals (scientific procedures) Act, 1986 as required by United Kingdom (UK) and European Union (EU). All work was performed under appropriate Biosafety Level 2 conditions (BSL2). All zebrafish care and experimental procedures were conducted according to Home Office legislation and the Animals (Scientific Procedures) Act 1986 (ASPA) under the Home Office project license 40/3681 and personal licenses l13220C2C to Kerstin Voelz and lCDB92D64 to Herbert Itabangi. Mouse studies were approved by the Institutional Animal Care and Use Committee of the Los Angeles Biomedical Research Institute at Harbor-UCLA Medical Center, according to the NIH guidelines for animal housing and care under protocol 11671.

### Method details

#### Generation of Conditioned medium

This was obtained by incubating at 4x10^9^ spores/mL in 250 mL of either serum free DMEM (for macrophage experiments) or HL5 medium (Formedium, for *D. discoideum* experiments) at 37^°^C with shaking at 200 rpm for 4 h. The medium was then centrifuged at 3990 rpm for 5 mins and the supernatant filter sterilised through a 0.45 μm filter.

Endosymbiont-free (cured) fungal strains were obtained by cultivating fungi in the presence of 60 μg/mL ciprofloxacin for a month. Fungi were then maintained on ciprofloxacin plates for 3 months before the endosymbiont was entirely cleared. Absence of bacteria was routinely confirmed through SYTO9 staining and PCR screening for bacterial 16S rDNA, as described below.

Spore stress resistance was measured by incubating at 10^5^ spores/mL in serum-free DMEM containing the indicated concentrations of hydrogen peroxide (H_2_O_2_), sodium nitrite (NaNO_2,_ Fisherscientific), sodium dodecyl sulphate (SDS) (Fisher scientific), sodium chloride (NaCl, Sigma-Aldrich) or Amphotericin B (AmB, Sigma Aldrich). The spores were incubated at 37°C and 5% CO_2_ for 24 or 48h before CFUs were determined by plating serial dilutions on SDA agar.

#### Reintroduction of *R. pickettii* in *R. microsporus*

Reintroduction of the endosymbiont was achieved as described previously, with some modifications.^43^ Cured *R. microsporus* spores were harvested and washed 3x with PBS before incubation at 37°C for 40 min with 30 mg/ml of lysing enzymes from *Trichoderma harzianum* (Sigma L1412) to genetrate protoplasts. Spores were then transferred in SCS buffer pH 5.8 (20mM sodium citrate, 20mM citric acid, 1M D-sorbitol) and co-cultured with *R. pickettii* on plates containing PDA. A plug of PDA was removed and replaced with a plug of LB agar covered with an inoculum of the bacteria. An inoculum of the fungus was placed directly on the bacteria and plates incubated at 30°C for 2 weeks.

To test for the presence of endobacteria, 12h-germinated spores were sheared by pipetting (first round 70x and a second round 20x) before centrifugation at first 32 *g* for 5 min, then 2,060 *g* for 20 min. After each centrifugation step, supernatant aliquots were plated onto Pseudomonas Agar® supplemented with 0.5% yeast extract and 1% glycerol (ThermoFisher Scientific CM0559) and incubated at 30°C.

At the same time, supernatants and cell pellet aliquots were retained for DNA isolation. Briefly, cell pellets and supernatants were disrupted using glass-beads and vortex (4 cycles, 1 min vortex:1 min ice) before snap freezing in liquid nitrogen before immediate transferrance to a water bath at 85°C for 20 min, before snap freezing again. DNA was then purified by phenol:chloroform:isoamyl alcohol (25:24:1) and ethanol precipitation. Primers amplifying the 16S rDNA of *Ralstonia* spp. were used. RalGS-F (5’CTGGGGTCGATGACGGTA3’) and RalGS-R (5’ATCTCTGCTTCGTTAGTGGC 3’) were used to identify the endosymbiont at the genus level (*Ralstonia* spp.) (amplicon 546 bp) and Rp-F (5’ATGATCTAGCTTGCTAGATTGAT 3’) and Rp-R (5’ACTGATCGTCGCCTTGGTG 3’) to identify it at species level (*R. pickettii*) (amplicon 210 bp).^108^^,^^119^^,^^120^ The PCR conditions as follows: initial denaturation for 4 min at 95°C, followed by 30 cycles at 95°C for 30 sec, 63°C for 40 sec, and 72°C for 35 sec, and a final extension at 72°C for 5 min. Primers Rm*ACT1*-F (5’GTCTTTCCTTCTATTGTTGGTC 3’) and Rm*ACT1*-R (5′-CCATCAGGAAGTTCATAAGAC-3′) were used to amplify actin gene from *R. microsporus* (amplicon 600 bp).^40^

#### Phylogenies

Trees were built based on 28S and 16S sequences respectively. Both were aligned with MUSCLE, bootstrapped and produced with RAxML.^117^^,^^118^ GenBank accession numbers for each sequence used were: *Ralstonia solanacearum* AB024604.1; *Candidatus Glomeribacter gigasporarum* AJ251633.1; *Mycetohabitans endofungorum* AM420302.1; *Ralstonia pickettii* 12J CP001068.1; *Ralstonia pickettii* 12D CP001644.1; *Paraburkholderia rhizoxinica* HKI 454 FR687359.1; *Rhizopus microsporus* CCF4531 HG324080.1; *Rhizopus microsporus* VPCI 128/P/10 KJ417570.1; *Rhizopus microsporus* var. *oligosporus* MH869766.1; *Rhizopus microsporus* var. *microsporus* MH870925.1; *Rhizopus microsporus* var. *chinensis* MH871901.1; *Rhizopus microsporus* var. *rhizopodiformis* MH873059.1

#### Cell wall analysis

For microscopy, 2x10^8^ spores/ml were incubated for 30 minutes at room temperature in PBS containing either 100 μg/ml fluorescein isothiocynate isomer 1 (FITC) (Sigma-Aldrich) in 0.1 M sodium bicarbonate buffer (pH 7.45) (Sigma Aldrich), 25-50 μg/mL RhTRITC-concanavalin A (ThermoFisher scientific) or 250 μg/ml calcofluor white (Sigma Aldrich). Spores were then imaged at 63x magnification on a Zeiss Axio Observer Z1 equipped with structured illumination (Apotome) using a Flash 4 sCMOS camera (Hamamatsu). The same exposure was used across all samples. Staining intensity was quantified for each cell using ImageJ to measure relative fluorescence within computationally identified regions of interest.

For flow cytometry, resting *R. microsporus* FP 469-12 spores were pre-germinated for the indicated times in serum-free DMEM at 37°C, 200 rpm, before fixation in 4% methanol-free formaldehyde, and incubation with either 0.01 μg/ml Dectin-1 IgG antibody, washed, and 1:200 goat anti-mouse IgG-488 secondary antibody (b-glucan); Calcofluor white (CFW, chitin; 250 μg/ml); or ConcanavalinA (ConA-488; 50 μg/ml). At least 10,000 cells were measured using an Attune NxT flow cytometer and compared to a pooled secondary-only control. Data are representative of three independent experiments.

#### TEM

*R. microsporus* spores were collected and allowed to swell as described above. Samples were processed via high-pressure freezing using a Bal-Tec HPM 010 high-pressure freezer (Boeckler Instruments, Tucson, AZ) and transferred to an RMC FS-7500 freeze substitution unit (Boeckler Instruments, Tucson, AZ) before substitution in 2% osmium tetroxide, 1% uranyl acetate, 1% methanol, and 5% water in acetone. Samples were then transitioned from −90°C to room temperature over 2 to 3 days, rinsed in acetone, and embedded in LX112 epoxy resin (Ladd Inc., Burlington, VT). 70 to 80 nm sections were then cut on a Leica Ultracut UC7 microtome, stained with uranyl acetate followed by lead citrate, and viewed on a JEOL 1200EX transmission electron microscope at 80 kV.

#### SYTO9 staining of bacterial endosymbionts

A small sample of mycelia pellet was aseptically submerged into 200 mL of 0.85% sodium chloride (NaCl) for 1 h, washed 2x in PBS and stained with the Live/Dead BacLight bacterial viability kit (Thermofisher Scientific). Mycelia were incubated in stain solution for 15 minutes, fixed with 4% PFA, mounted on glass slides and imaged using a 63x oil objective under phase contrast on a Zeiss Axio Observer Z1 microscope. For confocal imaging, 1x10^8^/ml spores in 10mls SabDex were incubated for 4 hrs, 150 rpm, then collected, washed 3x with sterile PBS, and stained with the Live/Dead BacLight bacterial viability kit (Thermofisher Scientific) according to the manufacturer’s instructions. Images were acquired on a Zeiss LSM 900 with Airyscan 2.

#### Screening and identification of bacterial endosymbiont

PCR screening for the presence of endosymbionts was performed using Universal primers (5’CCGAA TTCGTCGACAACAGAGTTTGA TCCTGGCTCAG3’/ 5’CCCGGGATCCAAGCTTACGGCTACCTTGTTACGACTT 3) which amplify a 1.5 kb 16S rDNA product.^106^ DNA was obtained from 10^7^ spores in a screw cup with beads and homogenised using a bead beater (Bertin technologies) at 6500x *g* for 1 min. DNA was then extracted with a DNeasy® powerlyzer® microbial kit (Qiagen) following the manufacturer’s instructions. PCR was performed using Phusion kit (New England Biolabs) and 35 cycles, of 95°C for 2 min, 60°C for 30s, and 72°C for 1 min. The resulting PCR product was then sequenced, identifying. *R. pickettii*. For full- genome sequencing, bacterial genomic DNA was extracted using the DNeasy PowerLyzer Microbial Kit (Qaigen) and sequenced by MicrobesNG (University of Birmingham, UK). The sample was identified by kraken (v1) as being highly similar to *R. picketti* J12.^111^ Transcriptional analysis of *R. pickettii* was assessed following growth for 4 h at 30 °C, 80-150 rpm in VK, DMEM, HL5, or DMEM+cured *R. microsporus* hyphae. Triplicate biological replicates were prepared for each condition. RNA was extracted using the modified Qiagen RNA extraction method as described previously.^121^ Briefly, TRIzol was used to lyse the samples, which were then either immediately frozen at -20°C and stored for RNA extraction or placed on ice for RNA extraction. After lysis, 0.2 ml of chloroform was added for every 1 ml of TRIzol. Samples were incubated for 3 min, then spun at 12,000 g at 4°C for 15 min. To the aqueous phase, an equal volume of 100% ethanol (EtOH) was added, before the samples were loaded onto RNeasy RNA extraction columns (Qiagen). The manufacturer’s instructions were followed from this point onwards. RNA quality was checked by Agilent, with all RNA integrity number (RIN) scores above 7. One microgram of total RNA was used for cDNA library preparation. Library preparation was done in accordance with the NEBNext pipeline, with libraries quality checked by Agilent. Samples were sequenced using the Illumina NextSeq platform; 150-bp paired-end sequencing was employed (2 x 150 bp) (>10 million reads per sample). Data was analyzed using Hisat2 (Version 2.0.5), HTSeq (Version 0.10.0) and edgeR (Version 3.16.5).^112^^,^^114^, ^115^, ^116^^,^^113^

#### Phagocytosis assays

For phagocytosis by macrophages, 1 x10^6^ J774A.1 cells were seeded per well a 24 well plate in DMEM and incubated overnight. Next day, cells were washed 2x in pre-warmed PBS, before incubation with 1 mL pre-warmed serum-free DMEM at 37^°^C +5% CO_2_ for 1 h. Cells were then washed 2x with PBS, before addition of 5x10^6^pre- FITC stained spores in serum-free DMEM (MOI = 1:5). The co-culture was incubated 37^°^C +5% CO_2_ for 1 h before fixation in 4% PFA for 15 min, and washing with PBS prior to counter staining with Concanavalin A (Con-A) or calcoflour white (CFW) for 30 min. Cells were then washed in PBS and imaged on a Nikon T1 microscope. Uptake rate was quantified as the number of phagocytes containing at least one spore, >1000 phagocytes assessed per replicate.

Phagocytosis of UV killed spores was performed as above, except prior to addition, spores were irradiating twice for 15 min in a UV PCL-crosslinker at 1200 μJ/cm^2^ in PBS, cooling on ice between treatments as previously described.^18^ Successful killing was confirmed by plating for CFUs. Latex beads, *C. albicans* SC5314 and *S. cerevisisae* AM13/001 were all processed similarly to spores including washing with PBS prior to addition to the macrophages.

Phagocytosis by *D. discoideum* was measured by incubating 2 x 10^6^ amoebae in 2ml untreated or conditioned HL5 in 3cm glass-bottomed microscopy dishes for 1 hour, prior to addition of 1 x 10^7^ TRITC-labelled heat killed *S. cerevisiae* (prepared as in^122^). After 30 minutes, fluorescence of unengulfed yeast was quenched by addition of 100 μl 0.4% trypan blue solution (Sigma Aldrich), before fluorescence microscopy and quantification of number of yeast engulfed per amoebae, counting >100 cells quantified per sample.

**Phagosomal proteolysis** was measured as previously described, using 3 μm silica beads co- labelled with DQ-green BSA and Alexa594 (performed as in^123^). Amoebae were washed twice in LoFlo medium (Formedium), before resuspension at 3 x 10^6^/ml in HL5 medium and 100 μl/ well seeded in clear-bottomed black-walled 96-well plates (Greiner). After 2 hours, 10 μl reporter beads were added to triplicate wells at a bead:cell ratio of 1:2, and the plate spun down at 1,200 rpm for 10 seconds to synchronise uptake. Free beads were then removed by tapping the inverted plate on a paper towel and washing twice in HL5. 100 μl conditioned, or control media was then added to wells before the plate was placed in a plate reader, and fluorescence measured at 500/520nm and 594/630nm (excitation/emission) every minute. For pre-treatment with conditioned medium, it was added 30 minutes prior to bead addition, and used in all subsequent washes/incubations. Proteolysis was calculated by the increase in DQ-BSA fluorescence over time, normalised to bead uptake, determined by Alexa594 fluorescence.

**Phagosomal killing** was performed as in^124^^,^^125^ following the quenching of GFP-expressing *Klebsiella pneumoniae* (gift from Pierre Cosson, University of Geneva). 10 μl of a saturated overnight bacterial culture in LB was diluted in 280 μl HL5 (or conditioned medium) and allowed to settle as a drop in a glass-bottomed microscopy dish (Mat-Tek). After 15 minutes, 1.5 ml of *D. discoideum* culture at 1 x 10^6^ cells/ml was carefully added and GFP and bright-field imaged recorded every 20 seconds for 40 minutes at 20x magnification. Movies were then manually analysed to determine the time of GFP- quenching post-engulfment.

#### Spore viability

Resistance to killing by macrophages was assessed as following phagocytosis at the indicated time points. Macrophages were lysed with 1 mL sterile water and aggressively washed to collect adherent spore cells. The lysate was serially diluted and 5 μL plated out on SAB agar for CFUs.

#### Zebrafish infections

Prior to injection, fungal spores collected from 10 day old cultures on SabDex plates were washed 3x in PBS and stained with Calcofluor White in 0.1 M of NaHCO_3_ for 30 min. Swollen spores were pre-incubated in DMEM at 37°C, 200 rpm for 4 hr prior to staining. Spores were the washed 3 times in PBS, and resuspended at 10^8^ spores/mL in 10% (w/v) polyvinylpyrrolidone-40 (PVP) in PBS with 0.05% phenol red in ddH_2_O. PVP was used as a carrier medium to increase density and prevent clogging of microinjection needles. Zebrafish were injected in accordance to a protocol by Brothers et al., and zebra fish development assessment in accordance to Kimmel et al..^126^^,^^127^ The fish were injected at prim-25 stage following manual dechorionation and anesthesia with 160 μg/mL of Tricaine in ddH_2_O. Micro-injection was performed with 2 nL of 10% (PVP) in PBS or *R. microsporus* spore suspension through the otic vesicle into the hind brain to achieve an inoculum dose of approximately 50-100 spores per fish. Following injection, larvae were anesthetized with 160 μg/mL Tricaine in E3 media in a 96 well plate and screened by fluorescence microscopy, (Zeiss Axioserver Zi microscope equipped with Apotome system) for the presence of spores. Only larvae with approximate correct inoculum were selected, and transferred to individual wells of a 24 well plate containing E3 media (plus 0.1% methylene blue ±60 μg/mL Ciprofloxacin). The fish were monitored over a period of 96 hours post infection for survival whereupon they were killed by 1600 μg/mL Tricaine overdose and treated with bleach overnight before disposal.

#### Recovery of fungal spores

Following injection, 5 fish per biological repeat were euthanized with 1600 μg/mL Tricaine at each timepoint, before homogenisation in 100 μL of E3 media containing penicillin-streptomycin (5000 U/mL-5 mg/mL) and gentamicin (10 mg/mL) using pellet pestles. Extracts were then plated out on SDA containing 100 U/mL-100 μg/mL penicillin-streptomycin and 30 μg/mL gentamicin, incubated at room temperature for between 24 and 48 h and colony forming units (CFUs) examined.

##### Phagocyte recruitment

To quantify phagocyte recruitment into the hindbrain, Tg(mpx: GFP)^i114^ or Tg(mpeg1:G/U:NfsB-mCherry) transgenic zebrafish^74^^,^^75^ were injected as above. At each timepoint, fish were imaged using an Axio Observer Z1 microscope equipped with Apotome (Carl Zeiss), to reconstruct the 3D volumes. Positive phagocyte recruitment was defined by accumulation of >10 neutrophils or macrophages to the site of infection. At least 3 biological repeats were performed with 5 fish per condition to give a total of 15 fish per group.

##### Mouse infections

Immunocompetent CD-1 20-23g male mice (n=12 per group) were randomly assigned to one of two treatment groups and the groups housed in IVCs. *Rhizopus microsporus* spores (FP469-12.6652333) were collected from Sabouraud agar plates in 10 mL of PBS + 0.01% Tween 20, washed once with endotoxin-free PBS and resuspended in serum-free DMEM at 4x10^8^/ml. To mimic the early stages of infection, swollen spores were pre-germinated in serum-free DMEM with or without Ciprofloxicin (60 μg/mL) for up to 3 hrs at 37°C, 200 rpm, sufficient to swell but not form germ tubes. Mice were infected intratracheally with 25μl serum-free DMEM containing 10^6^ resting or swollen spores. Mice (n=5 per group) were sacrificed by pentobarbital overdose and lungs were collected at two different time points: 4 h or 48 h post infection. Lungs were homogenization in 2 ml of PBS, and 200 μl were plated directly from the concentrated samples and also from serial dilutions onto potato dextrose agar + 0.1% Triton plates and incubated at 37 °C. Innoculum was verified via lung CFU from two mice directly after infection. Data were tested for normality using Shapiro-Wilk and analysed using the Mann-Whitney U test. Data are reported as Median with 95% CI.

### Quantification and statistical analysis

All data was analysed in Graphpad Prism 7 using the nonparametric, Mann- Whitney U tests, and log rank tests, with Dunn’s correction for multiple comparisons where appropriate and as indicated in the figure legends and main text. Differences with *p* value ≤0.05 were considered significant. All experiments were performed in triplicate, with the number of events measured indicated in figure legends. Data are plotted as Box-and- Whiskers with maximum and minimum values, except where data shown are percentages or relative values.

## Data Availability

Genomic sequencing data is available through the Sequence Read Archive (SRA) on NCBI and accession codes for sequences referenced in this work are: *R. pickettii*: SRA: SRR16523121 and *R. microsporus*: SRA: SRR16610381. This work did not lead to the generation of any new code.

## Supplemental information

Document S1. Figures S1–S6

Document S2. Article plus supplemental information
