# Supplementary material for: A bacterial endosymbiont of the fungus Rhizopus microsporus drives phagocyte evasion and opportunistic virulence
Source: Curr Biol. 2022 Mar 14;32(5):1115–1130.e6. doi: 10.1016/j.cub.2022.01.028 (PMC8926845; doi:10.1016/j.cub.2022.01.028)
Supplement: Document S1. Figures S1–S6 [file mmc1.pdf]

Current Biology, Volume *32*

Supplemental Information

A bacterial endosymbiont of the fungus *Rhizopus microsporus* drives phagocyte evasion and opportunistic virulence

Herbert Itabangi, Poppy C.S. Sephton-Clark, Diana P. Tamayo, Xin Zhou, Georgina P. Starling, Zamzam Mahamoud, Ignacio Insua, Mark Probert, Joao Correia, Patrick J. Moynihan, Teclegiorgis Gebremariam, Yiyou Gu, Ashraf S. Ibrahim, Gordon D. Brown, Jason S. King, Elizabeth R. Ballou, and Kerstin Voelz

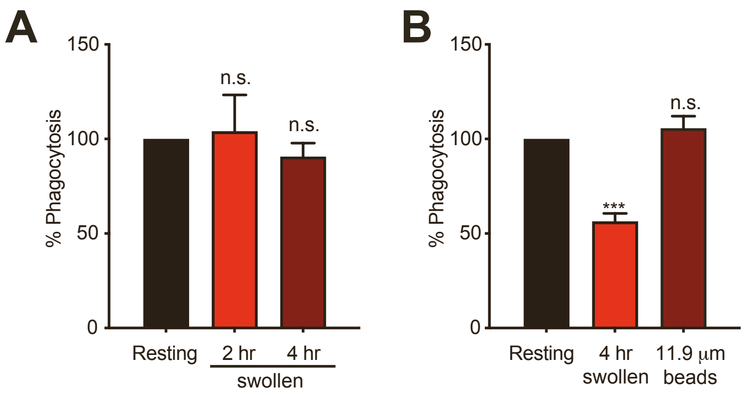

Figure S1: Inhibition of phagocytosis requires live spores and is not due to spore size, related to Figure 1. (A) Phagocytosis of R. microsporus FP 469-12 UV-inactivated resting and swollen (2 and 4hr) spores by J774A.1 macrophages. (B) Phagocytosis of swollen (4h, 6.5 μm) R. microsporus FP 469-12 spores and latex beads (11.9 μm) by J774A.1 macrophages. For both experiments the number of phagocytes containing at least one spore or bead were scored as positive. Graphs show mean ± SEM of 3 independent repeats. ns = p>0.05, ***=p<0.0001, One-Way ANOVA with Tukey's correction for multiple comparisons.

Done!

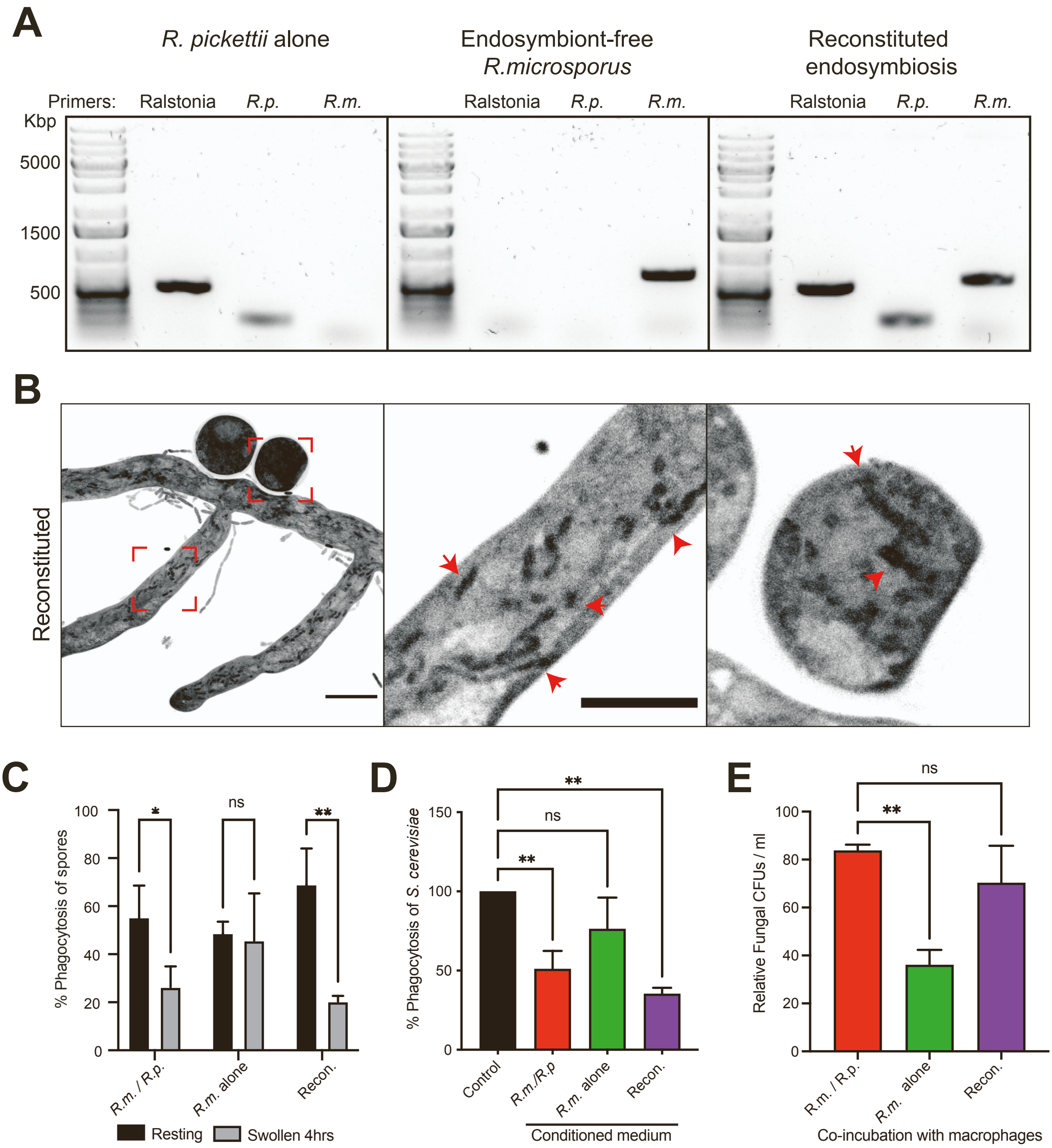

Figure S2: **Reconstitution of endosymbiosis restores anti-phagocyte activity, related to Figure 2** (A) PCR confirmation of endosymbiont clearance and reintroduction. PCR was performed on cell pellets of the cells indicated using 3 different primer pairs.The first colum shows PCR with Ralstonia genus primers (546bp), the second with *R. pickettii*-specific primers (210bp), and the third with *Rhizopus microsporus*-specific primers (600bp). (B) Confocal images of SYTO9 stained reconstituted spores. Left hand panel shows a maximum intensity projection (scale bar = 10 µm), boxed regions showing a spore and hyphae are enlarged in the right hand panels (scale bar = 5 µ m).(C) Phagocytosis of spores of endosymbiont-containing parental strain (R.m./R.p), endosymbiont-free strain (R.p.) and reconstituted endosymbiosis (Recon.) but J774.2 macrophages. (D) Inhibition of heat-killed *S. cerevisiae* phagocytosis by J774.2 macrophages in media conditioned by the same strains. (E) clearance of fungal spores by macrophages, measured by reduction in CFUs after 24 hours co-incubation. All graphs show the mean ± S.D. of 3 independent experiments. ** P<0.001, One way ANOVA with Tukey's correction for multiple comparisons.

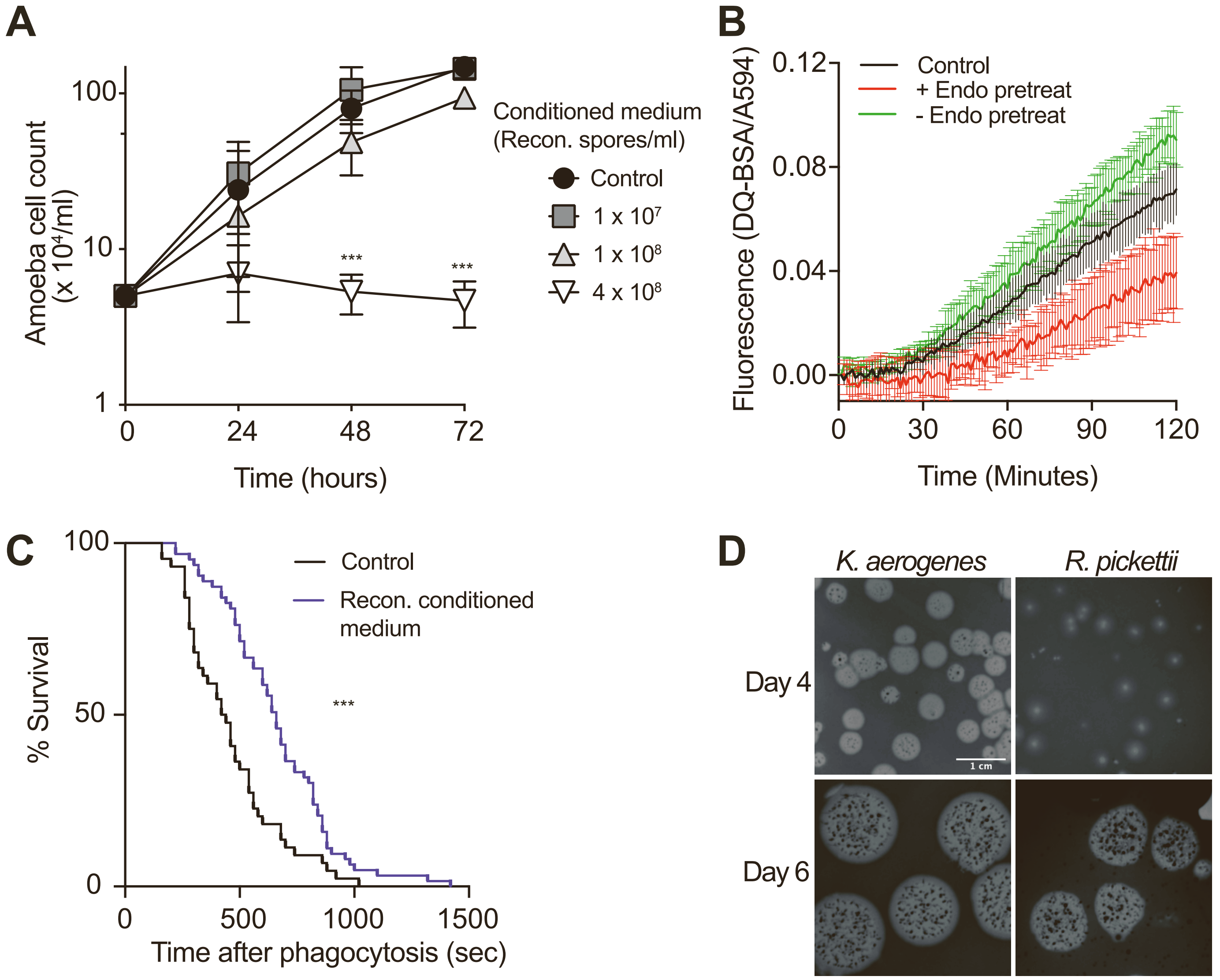

Figure S3: Reconstitution of endosymbiosis also restores activity against amoeba, related to Figure 3 (A) Effect of reintroduced endosymbionts on the ability of conditioned media to inhibit *D. discoideum* growth. Media was conditioned by the fungal spore densities for 4 hours prior to spore removal and addition to cultures of amoeba. (B) Pretreatment of *D. discoideum* with conditioned medium has no additional effect on phagosome proteolyis. Cells were pretreated with medium conditioned by R. microsporus FP 469-12 spores with and without endosymbionts for 30 minutes prior to addition of DQ-BSA proteolysis reporter beads. (C) Effect of media conditioned by 4 x 10^8 reconstituted endosymbiotic spores/ml on phagosomal killing, measured by survival of bacteria (GFP-*K. aerogenes*) after engulfment by *D. discoideum*.*** p<, Log-rank(Mantel-Cox) test.(D) Shows colonies of *D. discoideum* forming on lawns of either *K. aerogenes* or the isolated endosymbiotic *R. pickettii* strain.

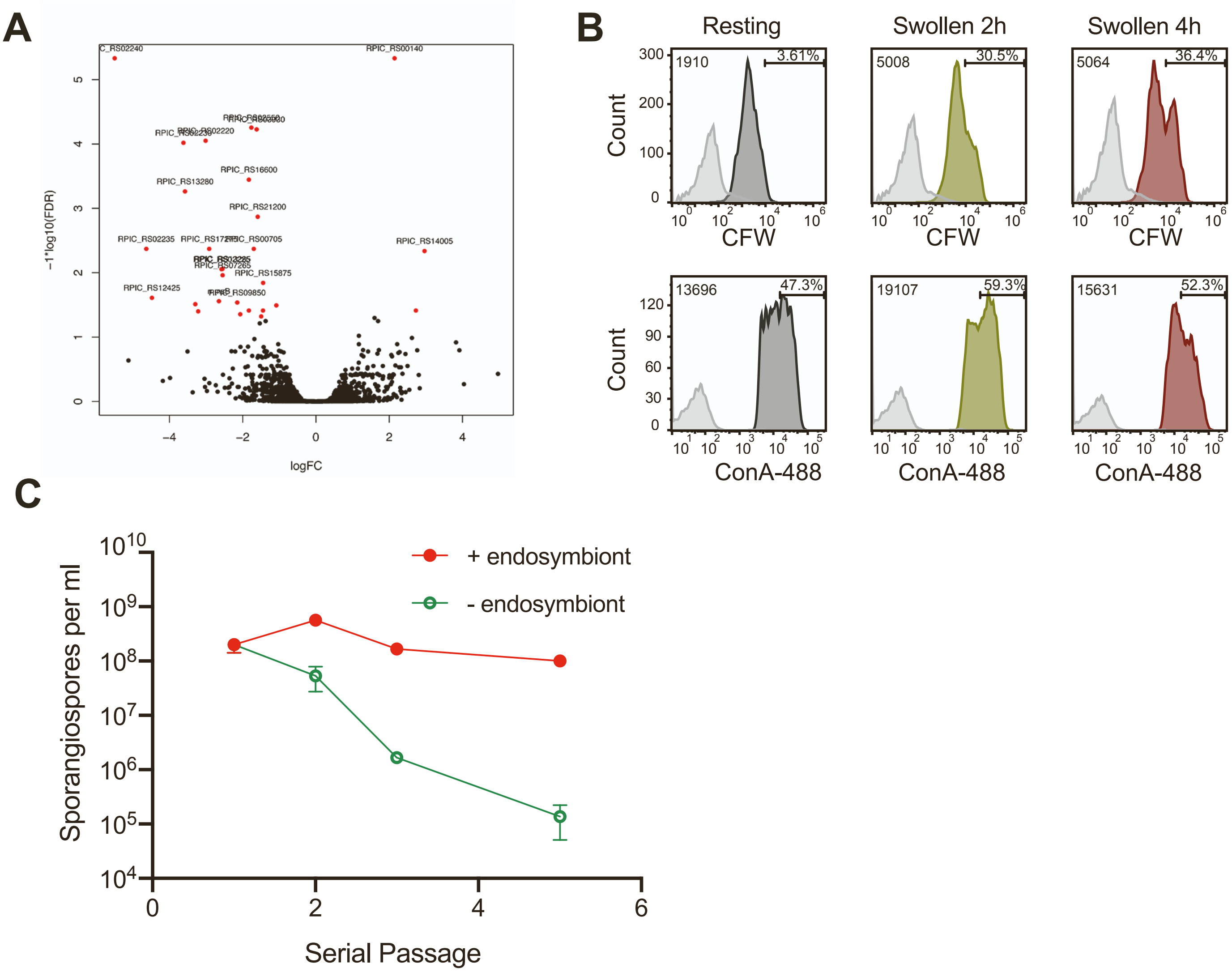

Figure S4: Endosymbionts affect host gene expression and cell wall composition, related to Figure 5. (A) Volcano plot comparing RNAseq data from R. pickettii across inducing (DMEM+fungus and HL-5) vs. non-inducing (VK and DMEM) conditions. Differentially expressed genes identified in red are those with an FDR of <0.001 and Log Fold Change ±2. RPIC_RS00140 encodes the unique T1PKS. (B) Total Chitin (CFW) and Mannan (ConA) exposure in resting and swollen spores analysed by flow cytometry (>10,000 cells), including Median Fluorescence Intensity (left) and Percent High (right) for each population. Data representative of three independent repeats is shown. (C) Sporangiospore counts for FP469-12 with and without the endosymbiont. FP469-12 was treated with 60ug/ml ciprofloxacin and serially passaged two times in the presence of ciprofloxacin. Sporangiospores were collected and frozen down into stocks. Holobiont and cured spores were cultured on SDA in the dark at 25C for 7 days and sporangiospores collected and counted by haemocytometer. Serial passage was performed 5 times, with triplicate plates.

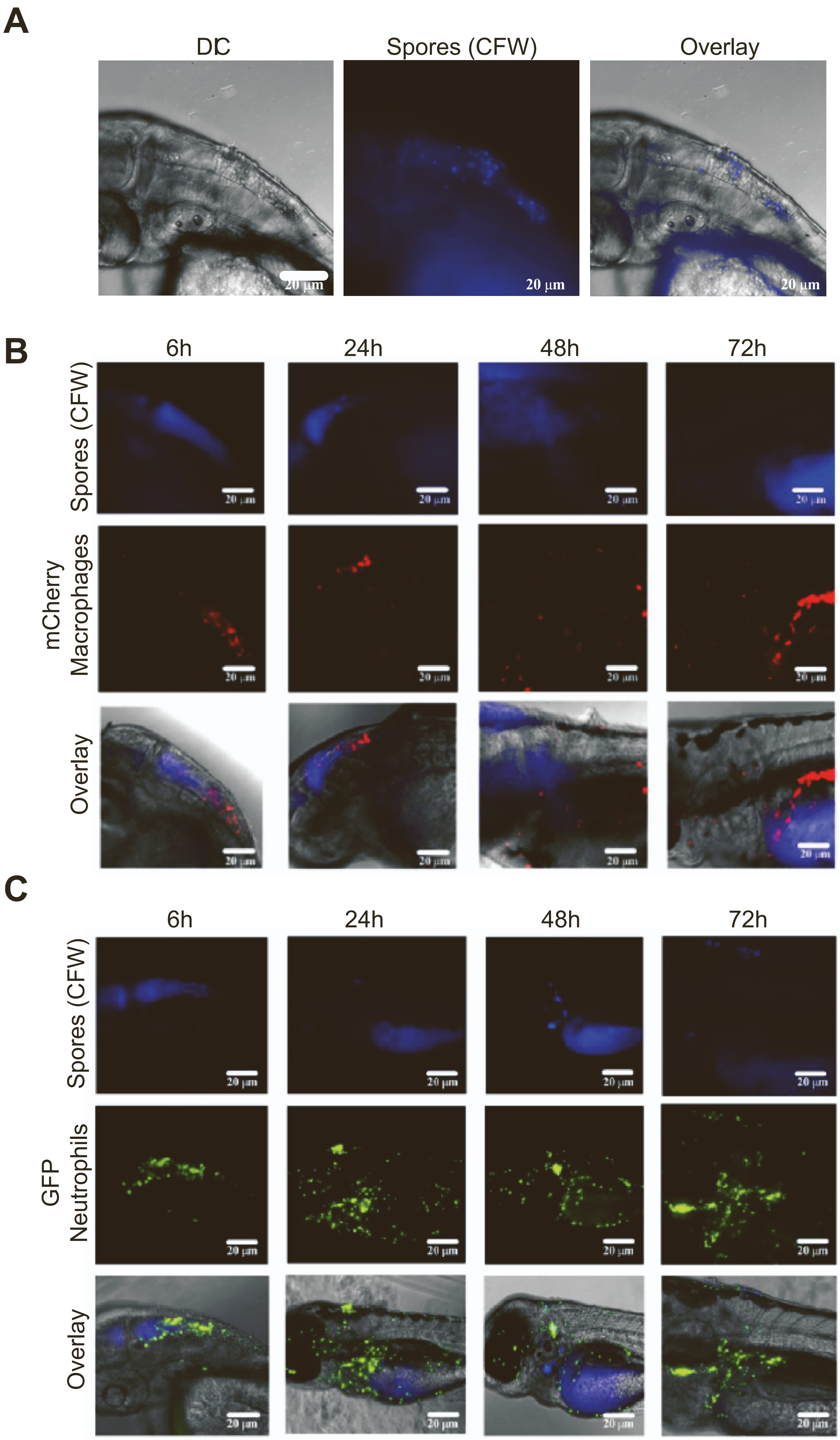

Figure S5: Zebrafish larval infection model, related to Figure 6. (A) Representative micrographs of zebrafish larvae infected with CFW-stained swollen parent spores as analysed in Figure 6. (B) Representative images of macrophage-reporter zebrafish (mpeg1:G/U:NfsB-mCherry) showing recruitment to sites of infection with CFW-stained fungal spores. (C) Equivalent images of infected GFP-neutrophil (mpx:GFP i114)-reporter fish.

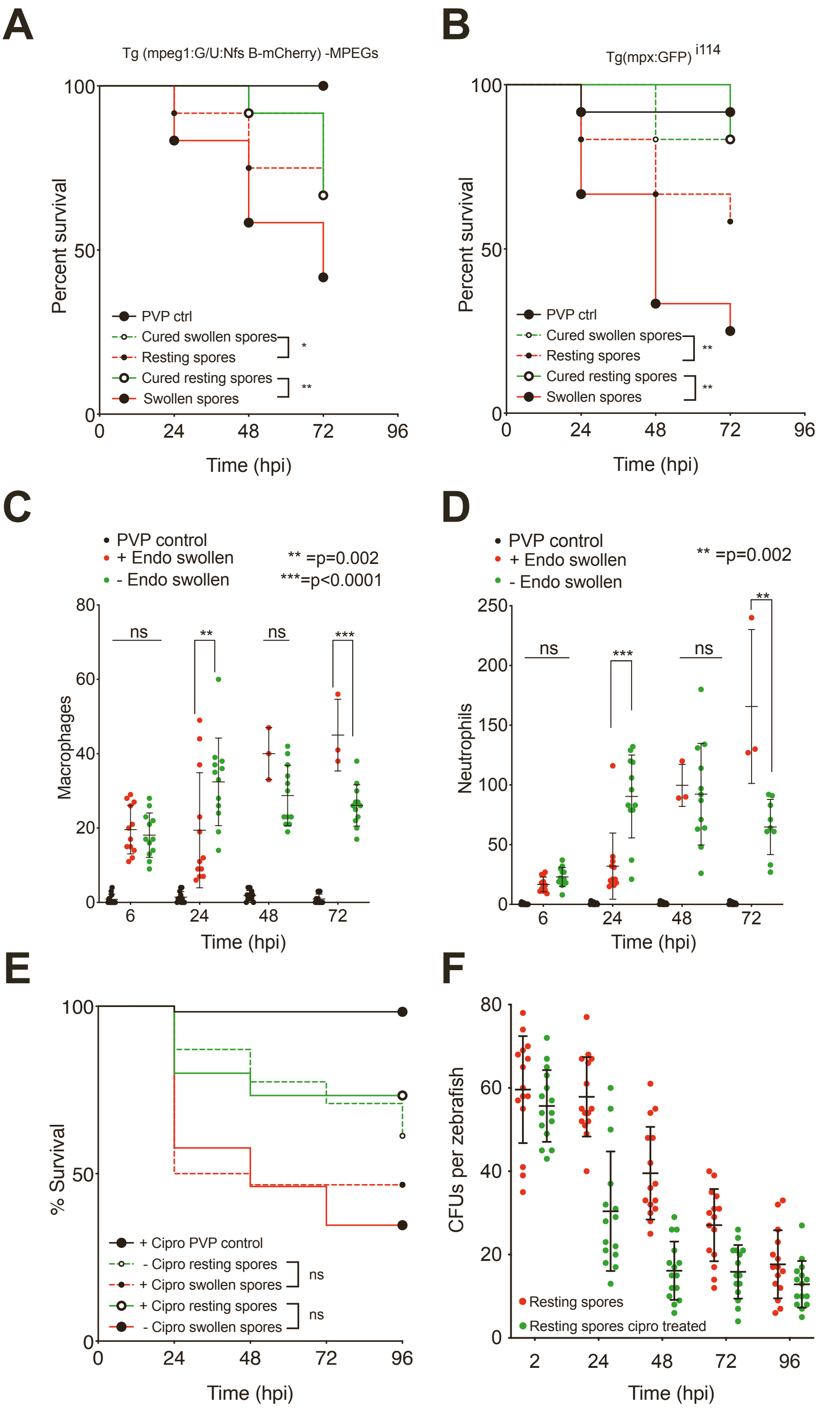

Figure S6: Effect of endosymbiont status on infection of zebrafish with swollen spores, related to Figure 6. (A) Kaplan-Meijer survival curves of transgenic macrophage-reporter (Tg(mpeg1:G/U:NfsB-mCherry)) zebrafish following hindbrain injection with R. microsporus FP 469-12 spores with and without endosymbionts. (B) Equivalent survival data of neutrophil-reporter (Tg(mpx:GFP)i114) zebrafish. n=12 fish per condition, statistical differences were determined using Mantel-Cox with Bonferroni's correction for multiple comparisons (5% family-wise significance threshold = 0.025). (C) and (D) Effect of infection with pre-swollen spores (4hr) on macrophage and neutrophil recruitment respectively. Three biological replicates of 4 fish per condition were examined (n=12). Statistical significance was assessed by Two-way ANOVA with Tukey's correction for multiple comparisons or pairwise t-tests where sample number was unequal due to fish death. (E) Survival curves for impact of 60 µg/ml ciprofloxacin on survival of wildtype AB fish upon hindbrain infection with resting or swollen parental R. microsporus FP 469-12 spores (n=30). Injected fish were then cultivated in E3 medium with or without 60 µg/ml Ciprofloxacin and monitored for mortality (n=30). Statistical differences were determined using Mantel-Cox with Bonferroni's correction for multiple comparisons (5% family-wise significance threshold = 0.025). (F) Shows CFU's recovered from fish treated as in (E). No statistical significance was observed at any time point. In all panels *=p<0.05; **=p<0.001; ***=p<0.0001 unless otherwise indicated.
